# Antiviral and immune modulatory activities of STING agonists in a mouse model of persistent hepatitis B virus infection

**DOI:** 10.1371/journal.ppat.1013709

**Published:** 2025-12-09

**Authors:** Ya Wang, Shuo Wu, Xingqiong Li, Lijun Qiao, Huiqiang Wang, Ge Yang, Haiyan Yan, Kun Wang, Jian-Dong Jiang, Yuhuan Li

**Affiliations:** 1 CAMS Key Laboratory of Antiviral Drug Research, Beijing Key Laboratory of Technology and Application for Anti-Infective New Drugs Research and Development, NHC Key Laboratory of Biotechnology for Microbial Drugs, Institute of Medicinal Biotechnology, Chinese Academy of Medical Sciences and Peking Union Medical College, Beijing, China; 2 State Key Laboratory of Bioactive Substance and Function of Natural Medicines, Institute of Medicinal Biotechnology, Chinese Academy of Medical Sciences and Peking Union Medical College, Beijing, China; University of Pittsburgh School of Medicine, UNITED STATES OF AMERICA

## Abstract

Activation of antiviral immune responses against hepatitis B virus (HBV) is essential for the durable control of chronic HBV infection and the functional cure of chronic hepatitis B. As a molecular hub at the interface of innate and adaptive immunity, the stimulator of interferon (IFN) genes (STING) is well suited as a therapeutic target to break the immune tolerance against chronic viral infections and tumors. Using STING knockout and human STING knock-in mouse models, we first demonstrated that STING agonist treatment activated robust innate immune responses in the spleen and liver and efficiently suppressed HBV DNA replication in the livers of AAV-HBV transduced mice in a STING-dependent manner. We further demonstrate that AAV-HBV transduced mice are well tolerated for the long-term treatment of STING agonist diamidobenzimidazole (diABZI) at the optimized dose and dosing schedules. Virological and hepatic bulk and single-cell RNA-seq analyses revealed that diABZI treatment activated immune responses in liver microenvironment and significantly inhibited HBV replication and HBeAg expression. Antibodies against HBV surface antigen (HBsAg), HBs-Ab, became detectable in 1/4 mice after 5 weeks of treatment. In conclusion, our findings imply that diABZI treatment activates a STING-dependent immune response that controls HBV replication in a mouse model of persistent HBV infection and thus establishes a scientific basis for further development of STING agonists as immune therapeutics for the treatment of chronic hepatitis B.

## Author summary

Although the combination therapy of viral DNA polymerase inhibitors and siRNA suppresses HBV load to undetectable levels and reduces serum HBsAg by more than 100-fold, the functional cure of chronic HBV infection still cannot achieve in most treated patients. The reason is that the treatment fails to restore functional antiviral immune responses that durably control the replication of residual viruses. Among many immunotherapeutic options, the tolerability and efficacy of STING agonist therapy for chronic hepatitis B has not been evaluated in biologically relevant animal models. The work reported herein demonstrates for the first time that the long-term STING agonist treatment is tolerable and induces antiviral immune responses that control HBV replication. Further investigation on the optimization of STING agonists and therapeutic strategies is warranted.

## Introduction

Chronic hepatitis B virus (HBV) infection remains an enormous public health burden that afflicts 296 million people worldwide and results in 880,000 deaths annually from end-stage liver diseases [1]. The current standard-of-care medications for chronic hepatitis B (CHB) are nucleos(t)ide analogue (NA) viral DNA polymerase inhibitors and pegylated interferon alpha (PEG-IFN-α) that modulate host antiviral immune responses. Although inhibition of HBV replication with long-term NA therapy is associated with improvement of liver diseases and reduction of HCC morbidity and mortality, the functional cure of chronic HBV infection, as indicated by the seroclearance of HBV surface antigen (HBsAg) is rarely achieved [2]. Moreover, although recent clinical trials demonstrated that combination therapy of NA and novel direct-acting antivirals against HBV in clinical development, such as capsid assembly modulators (vebicorvir or JNJ-56136379) and/or liver-targeting HBV siRNA, more profoundly suppressed HBV replication [3–8], the increased inhibition of HBV replication by the combination therapies cannot eliminate or inactivate the covalently closed circular DNA (cccDNA) and integrated HBV DNA in hepatocytes, the reservoirs of HBV replication and HBsAg expression [9,10]. Therefore, in addition to viral replication inhibitors, drugs that can activate a functional antiviral immune response against HBV are required to induce a sustained immune control of the residual HBV infection and achieve the functional cure of CHB [11–14].

Several immunotherapeutic approaches are currently in preclinical and clinical development for the functional cure of CHB. *First*, reconstitution of antiviral immune response *via* adoptive transfer of engineered HBV-specific T cells, such as T cells expressing chimeric antigen receptor (CAR-T) against HBV [15–18] or endogenous T cells transiently expressing HBV-specific T cell receptors (TCRs) through nonviral gene transfer technology, has been shown to transiently control HBV replication in humanized mouse models [19–21] and in human clinical trials [22,23]. *Second*, restoration of exhausted T cell function in chronic HBV carriers has been attempted by T cell checkpoint blockade therapies [24,25]. A phase 1b clinical trial of PD1-PDL1 blockade with nivolumab in HBeAg negative CHB patients showed a modest decline of HBsAg in most treated patients, while one patient achieved a sustained HBsAg seroclearance [26,27]. A recent clinical study reveals that the immune checkpoint inhibitor therapy accelerates HBsAg seroclearance in patients with cancer and baseline HBsAg less than 100 IU/ml [28]. *Third*, considering the important role of proinflammatory cytokines and innate immune cells in the induction of adaptive antiviral immune response to control HBV infection [29–32], therapies with the agonists of pattern recognition receptors (PRRs), particularly TLR7 and TLR8, have been explored to induce an effective immune control of chronic HBV infection [33,34]. Particularly, treatment of WHV-chronically infected woodchucks with TLR7 agonists (GS-9620, RG7854 and JNJ-64794964) or TLR8 agonist (GS-9688) activated intrahepatic CD8^+^ T cells, NK cells, B cells and interferon response transcriptional signatures and induced a sustained suppression of WHV replication, loss of WHBsAg, and more strikingly, sustained antibody response against WHBsAg in a subset of animals [35–37]. However, only limited efficacy was observed in clinical trials, most likely due to the low tolerability to the therapies [38–42].

Stimulator of interferon genes (STING) is an integral endoplasmic reticulum (ER) membrane protein that can be activated by guanosine-adenosine 2’,3’-cyclic monophosphate (2’3’-cGAMP) synthesized by cyclic guanosine monophosphate-adenosine monophosphate synthase (cGAS) upon activation by double-stranded DNA [43,44]. STING is expressed in macrophages, dendritic cells and lymphocytes, and plays an important role in the control of viral infections through activation of innate and adaptive immune responses [45–47]. As a molecular hub at the interface of innate and adaptive immunity, STING is well suited as a therapeutic target for breaking immune tolerance to chronic viral infections and tumors. Accordingly, many chemotypes of STING agonists have been discovered in recent years and developed as vaccine adjuvants and/or therapeutics for cancer and viral infections [33,48]. Thus far, it has been shown that activation of STING in human hepatoma cells suppresses HBV cccDNA transcription [49]. STING agonist treatment also promotes dendritic cell maturation and antigen presentation [50]. Moreover, treatment with murine STING agonist dimethylxanthone acetic acid (DMXAA) suppressed HBV replication in the livers of mice and attenuated the severity of liver injury and fibrosis in a recombinant cccDNA mouse model [51,52]. Diamidobenzimidazole (diABZI) is a potent activator of both murine and human STING with superior pharmacological properties and anti-tumor and antiviral activities *in vivo* in animal models [53–55]. We report herein the evaluation of the tolerability and therapeutic potential of STING agonists for chronic hepatitis B in AAV-HBV transduced mice. Our findings suggest that diABZI treatment induces a STING-dependent immune response in the liver microenvironment and controls HBV replication.

## Results

### DiABZI treatment induces a robust cytokine response and modulates immune cell function in the livers and spleens of mice

**Fig 1:**
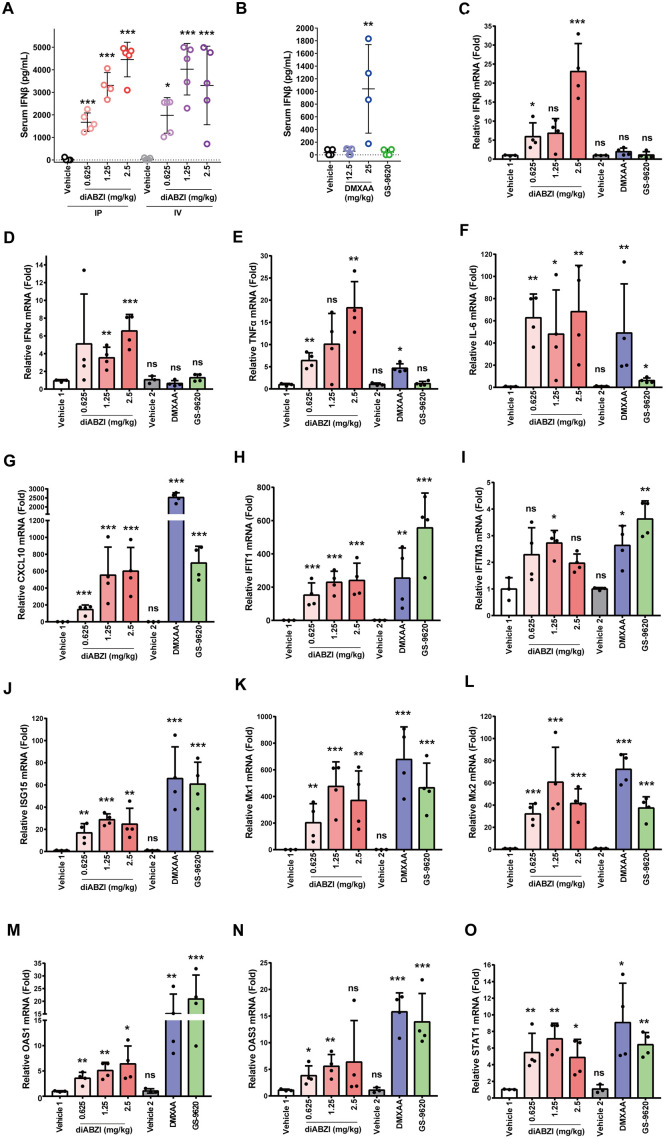
DiABZI activates a robust innate immune response in mice. **(A-B)** C57BL/6J male mice were treated with diABZI (0.625, 1.25 and 2.5 mg/kg) or vehicle by either intraperitoneal (IP) injection or tail intravenous (IV) injection **(A)**. C57BL/6J mice were treated with GS-9620 (15 mg/kg) by gavage and DMXAA (12.5 and 25 mg/kg) or vehicle by intraperitoneal (IP) injection **(B)**. At 4 h after treatment, the serum levels of IFNβ were determined by ELISA (n = 4-5/group). ***P < 0.001,**P < 0.01, *P < 0.05 by One-way ANOVA. **(C-O)** C57BL/6J male mice were treated with diABZI (0.625, 1.25 and 2.5 mg/kg), DMXAA (20 mg/kg) or vehicle by IP injection, or GS-9620 (15 mg/kg) by gavage and sacrificed at 2 h after treatment. The mRNA levels of IFNβ, IFNα, TNFα, IL-6, CXCL10, IFIT1, IFITM3, ISG15, Mx1, Mx2, OAS1, OAS3 and STAT1 in the livers were determined by qRT-PCR (normalized to GAPDH) (n = 3-4/group). Fold induction of gene expression relative to that in vehicle-treated controls was presented as mean values ± standard deviations (SD). With the exception of IFITM3, the others were subjected to a natural logarithm transformation (ln transformation), and then a One-way ANOVA analysis was performed. ***P < 0.001,**P < 0.01, *P < 0.05.

The expression of STING is usually restricted to the endoplasmic reticulum (ER) membrane of macrophages, T lymphocytes, dendritic cells and cells in respiratory tract and some other tissues (Tissue expression of STING1 - Summary - The Human Protein Atlas). Although hepatocytes express low levels of STING [56], it had been demonstrated that activation of STING in macrophages induced a robust cytokine response that effectively suppressed HBV replication in hepatocytes [51]. Considering the importance of cytokine response for immune control of HBV infection [30,31], we first determined the property of diABZI induced innate immune response in mice, as compared to mouse specific STING agonist DMXAA and TLR7 agonist GS-9620 [57,58]. Interestingly, IFN-β was detectable in the serum of mice treated with diABZI or DMXAA in a dose-dependent manner, but not in the serum of mice treated with GS-9620 at 4 h post treatment (Fig 1A and 1B). Consistent with the robust systematic IFN-β response observed in serum, diABZI treatment increased the levels of IFN-β, IFN-α, TNF-α, IL-6 and CXCL10 mRNA in liver tissues (Fig 1C to 1G). However, DMXAA treatment increased the levels of TNF-α, IL-6 and CXCL10 mRNA, but not IFN-β and IFN-α mRNA in liver tissues. Interestingly, GS-9620 treatment failed to induce the expression of all the cytokines examined, except for CXCL10. Despite the striking difference in hepatic cytokine induction profiles, the three compounds induced the expression of all the IFN-stimulated genes (ISGs) examined in the liver of mice (Fig 1H to 1O). While the difference in hepatic cytokine induction profiles between diABZI and GS-9620 could be due to the distinct expression of STING and TLR7 in liver cells, the difference between diABZI and DMXAA might be due to their distinct engagement with mouse STING and/or difference in pharmacological properties.

To further evaluate the regulatory effects of diABZI on immune cells, C57BL/6J male mice were treated with 0.31 mg/kg of diABZI or vehicle by intraperitoneal (IP) injection. At 16 h after treatment, the immune cells in spleens were analyzed by flow cytometry. The results showed that diABZI treatment did not significantly alter the total number of monocytes in spleens, but increased the proportion of neutrophils, macrophages, and dendritic cells (DCs) (S1A Fig, panels a to d). Moreover, diABZI treatment induced macrophage activation and differentiation towards M1 phenotype (S1A Fig, panels e to f), promoted the maturation of myeloid DCs (CD80^+^), but not plasmacytoid DCs (pDCs) (S1A Fig, panels g to h). In addition, diABZI treatment also promoted the activation of CD4^+^ and CD8^+^ T cells (CD69^+^) without altering their proportions (S1B and S1C Fig). A moderate increase in Th17 and Treg subsets was also observed (S1B Fig). Impressively, diABZI treatment strongly activated the effector function of T cells and NK cells, as evidenced by the significant upregulation in IFNγ^+^ and Granzyme B^+^CD8^+^ T and NK cells (S1C to S1E Fig). Finally, activation of STING and down-stream signaling pathway in splenocytes by diABZI or DMXAA was confirmed by immunoblotting detection of phosphorylated STING, TBK1, IRF3 and p65 (RelA) (S1F Fig). In summary, the results presented above indicate that the activation of STING by single-dosing diABZI treatment induces a robust innate cytokine response in the liver and strongly promotes myeloid and lymphocyte activation in the spleen.

### The immune modulatory and anti-HBV activities of diABZI and DMXAA in mice depend on species-specific STING expression

To verify whether the immune modulatory activity of diABZI and DMXAA is STING-dependent, splenocytes harvested from WT and STING knockout (STING^-/-^) mice were mock-treated or treated with three different STING agonists. Lack of STING expression in the knockout mice was demonstrated by Western blot assay in splenocytes from STING^-/-^ mice (S2A Fig). As anticipated, each of the three STING agonists, DMXAA, diABZI and MSA-2, induced robust expression of inflammatory cytokine (IL-6), chemokine (CXCL10) and ISGs (IFIT1, ISG15, Mx1 and MX2) in splenocytes derived from WT, but not STING^-/-^ mice (S2B Fig).

**Fig 2:**
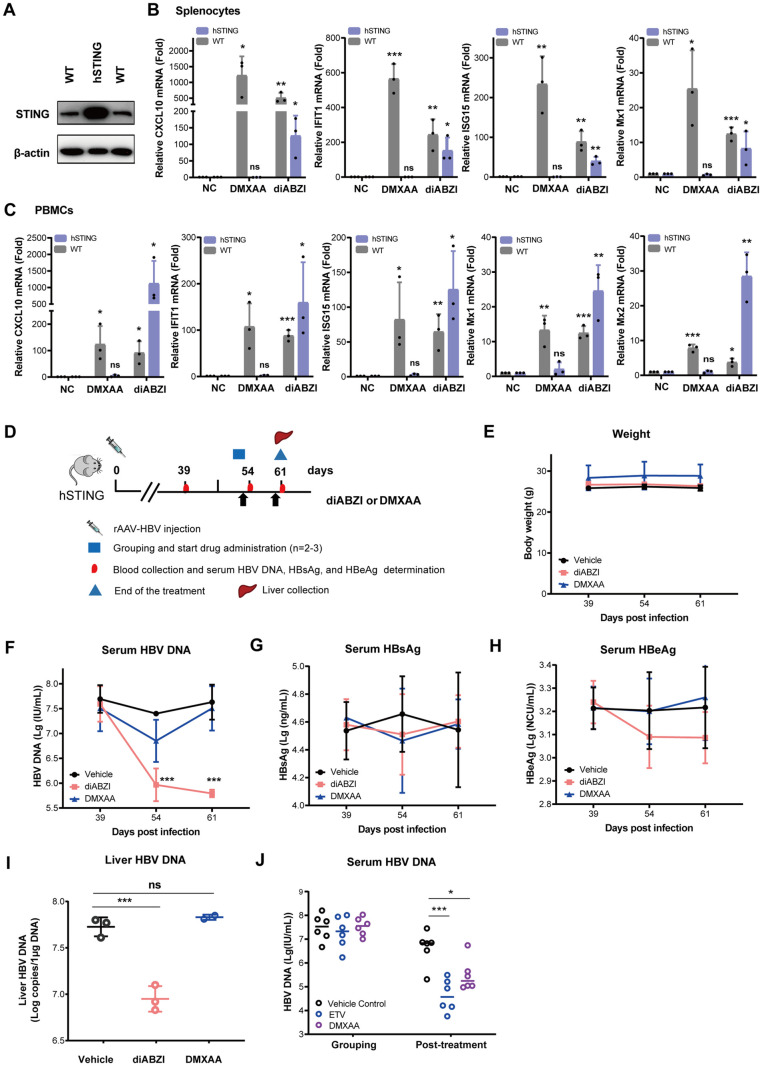
Effect of diABZI treatment on HBV replication in rAAV-HBV transduced STING humanized (hSTING) mice. **(A)** The STING protein level in the spleen of hSTING mice was detected by a Western blotting assay with an anti-STING antibody which can detect both human and mouse STING. **(B-C)** Effect of DMXAA and diABZI on the expression of CXCL10, IFIT1, ISG15, Mx1 and Mx2 in splenocytes and PBMCs from hSTING mice. The amounts of mRNAs specifying the specific cytokines and ISGs were quantified by qRT-PCR assay. Data are expressed as fold induction of gene expression relative to that in negative control (NC) treated with DMSO. ***P < 0.001,**P < 0.01, *P < 0.05 by Student’s *t*-test. **(D-I)** Experimental setup for *in vivo* efficacy study in humanized STING mice **(D).** All mice received a single i.v. injection of 8 × 10^10^ vg of rAAV8-HBV1.3. On day 52 after inoculation, mice were grouped and treated with 2 mg/kg of diABZI or 20 mg/kg of DMXAA. Mice treated with vehicles served as control. Images of the mouse, syringe, and liver were sourced from https://openclipart.org/17558, 282069, and 37315, respectively. **(E)** Body weight of mice before and after drug treatment is presented. **(F-H)** Serum levels of HBV DNA, HBsAg and HBeAg were determined by qPCR or ELISA on days 39 (before treatment), 54, and 61 after rAAV-HBV injection. ^***^P < 0.001 by Two-way ANOVA. **(I)** Liver HBV DNA levels were determined by qPCR (normalized per 1 μg of total liver DNA).^***^P < 0.001 by One-way ANOVA. **(J)** Effect of DMXAA and ETV on serum levels of HBV DNA in the rAAV-HBV transduced WT mice. C57BL6/J mice injected with 8 × 10^10^ vg of rAAV8-HBV1.3 genotype D by the tail vein for 8 weeks were divided into three groups (n = 6/group). Mice were then administered with a single dose of vehicle or DMXAA (25 mg/kg) via IP. Mice were also given ETV (0.1 mg/kg) by gavage daily. After one week posttreatment, the levels of serum HBV DNA in three groups of mice were measured by qPCR assay.***P < 0.001,*P < 0.05 by One-way ANOVA.

Because some of the non-cyclic dinucleotide STING agonists are human STING specific, their biological activities cannot be evaluated in mice [59]. To overcome this problem, the human STING gene had been knocked into the loci of mouse STING and created a humanized STING (hSTING) mouse line (Fig 2A). To validate the utility of the hSTING mice in pharmacological study of STING agonists, we first compared the activity of diABZI and mouse STING-specific agonist DMXAA in splenocytes and peripheral blood mononuclear cells (PBMCs) obtained from WT and hSTING knock-in mice. As shown in Fig 2B and 2C, while DMXAA only induced CXCL10 and ISG expression in splenocytes and PBMCs from WT mice, diABZI treatment induced the expression of CXCL10 and ISGs in splenocytes and PBMCs from both WT and hSTING knock-in mice. Interestingly, diABZI induced much higher levels of CXCL10 and ISGs mRNA in PBMCs, but not in splenocytes derived from hSTING mice. The reason for this discrepancy is possibly due to the higher basal level of STING expression in hSTING knock-in mice (Fig 2A).

To investigate the antiviral activity of STING agonist-induced innate immune responses against HBV in mice, WT C57BL/6J mice, hSTING mice or STING-KO mice were transduced by recombinant AAV-HBV particles and treated with diABZI or DMXAA with the dosing schedule specified in Figs 2D and S2C. As shown in Fig 2E to 2I, while treatment of AAV-HBV transduced hSTING mice with diABZI significantly reduced serum HBV DNA as well as intrahepatic HBV DNA, DMXAA treatment did not significantly alter any of the HBV markers examined. However, in agreement with previous reports [51,52], DMXAA treatment of AAV-HBV transduced WT mice significantly reduced the level of serum HBV DNA (Fig 2J). As anticipated, diABZI treatment of AAV-HBV transduced STING^-/-^ mice failed to significantly alter the levels of serum HBV DNA, HBsAg and HBeAg as well as intrahepatic HBV DNA and pgRNA (S2D-H Fig). These results clearly indicate that STING agonist treatment of mice induces robust innate immune responses in the livers and spleens and significantly suppresses HBV replication and HBeAg expression in a STING-dependent manner. However, STING agonist-induced immune response failed to reduce the levels of serum HBsAg during the short-term treatment (Fig 2G).

### Prolonged diABZI treatment suppresses HBV replication and HBeAg expression in AAV-HBV transduced mice

To activate an antiviral immune response for the durable control of chronic HBV infection, we speculated that long-term, multiple doses of STING agonist treatment ought to be required. Therefore, we first determined the tolerable dose and dosing strategy of diABZI in mice. As shown in S3 Fig, our initial experiment revealed that single IP or IV injection of diABZI at 0.625 mg/kg or higher doses induced weight loss of mice between day 1 to day 3 after injection. Similar weight loss was also observed in mice after a single IP injection of DMXAA at 25 mg/kg [51]. Further experiments with reduced dosing levels of diABZI or DMXAA showed that IP injection of 0.31 mg/kg diABZI or 20 mg/kg DMXAA every four days for 5 doses did not significantly change the body weight of mice (S4A Fig). To identify the safe dosing strategy to support a two-month STING agonist therapy, we examined further reduced dosing levels of STING agonists and found that treatment of mice with diABZI at 0.08 or 0.16 mg/kg and DMXAA at 10 or 15 mg/kg with the indicated dosing frequency for 72 days did not significantly change body weight of mice (S4B Fig), liver histology (S4C Fig), serum ALT and Cre levels (S4D and S4E Fig). Moreover, we confirmed that single IP injection of diABZI at 0.04, 0.08, 0.16 or 0.31 mg/kg, or DMXAA at 10 or 15 mg/kg activated innate immune response in the liver, as indicated by the significant induction of ISGs expression in liver tissues (S5 Fig). Importantly, 0.31 mg/kg diABZI exhibited significant antiviral activity by markedly reducing HBV DNA levels in the serum and liver in a hydrodynamic injection (HDI) mouse model of HBV (S6 Fig).

**Fig 3:**
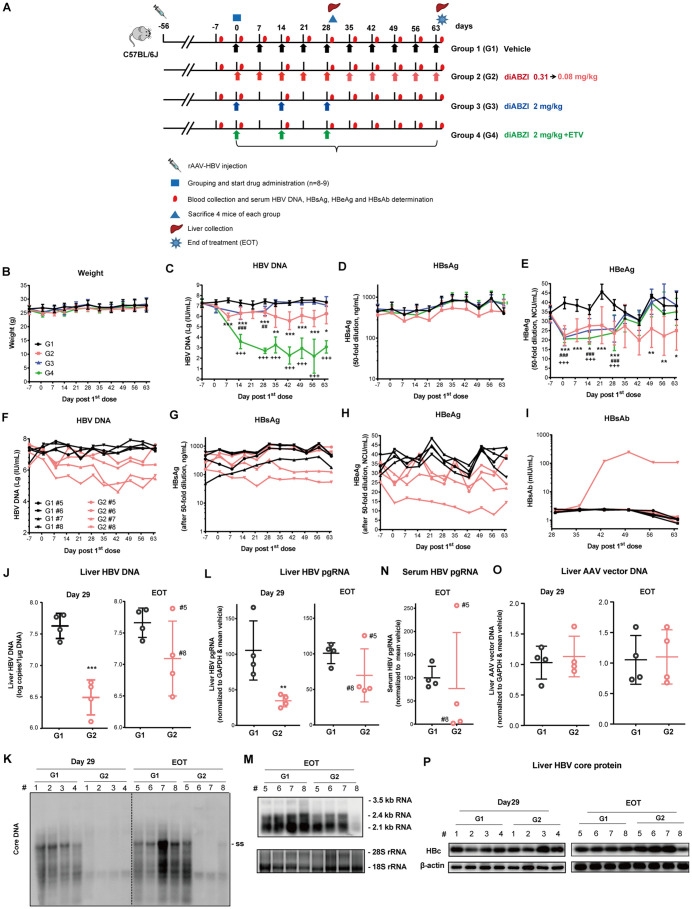
Effects of diABZI treatment on HBV replication and humoral immune response in the rAAV-HBV infected mice. **(A)** Schematic illustration of experimental schedule. Briefly, 6-week-old male C57BL6/J mice were transduced with 8 × 10^10^ vg of rAAV8-HBV1.3 genotype D by tail vein injection. Eight weeks later, the transduced mice were divided into four groups (n = 8-9/group) and dosed with vehicle (Group 1) or diABZI by IP injection at the indicated times, alone (Group 2 and Group 3) or in combination with ETV (0.1 mg/kg, once daily) (Group 4) by gavage. Mice were euthanized on day 29 after treatment (n = 4/group) or at end of treatment (next day after last dose) (n = 4-5/group). Body weight and virological markers were measured at the indicated time points. Day 0 corresponds to the start of dosing. Images of the mouse, syringe, and liver were sourced from https://openclipart.org/17558, 282069, and 37315, respectively. **(B-E)** The mice body weight, serum levels of HBV DNA, HBsAg and HBeAg in the four groups were determined. Mean values ± SD are plotted for each group. ^*^P < 0.05,^**^P < 0.01,^***^P < 0.001, Group 2 *vs* Group 1; ^##^*P* < 0.01,^###^*P* < 0.001, Group 3 *vs* Group 1; ^+++^*P* < 0.001, Group 4 *vs* Group 1 by Two-way ANOVA. **(F-H)** Dynamic levels of HBV DNA, HBsAg, and HBeAg from day -7 to the end of the experiment for each of mice in Group 1 and Group 2 are presented. **(I)** Dynamic levels of HBsAb during day 29-64 for each of the mice in Group 1 and Group 2 are presented. **(J-K)** Intrahepatic HBV DNA load was determined by qPCR and presented as log copies per 1 μg of total liver DNA or by Southern blot hybridization, respectively. ss, single-stranded DNA. **(L-M)** The levels of pgRNA in the liver tissues were quantified by qRT-PCR (normalized to GAPDH) or by Northern blot hybridization, respectively. **(N) The** serum levels of HBV pgRNA were quantified by qRT-PCR. **(O)** The levels of AAV vector DNA were quantified by PCR (normalized to GAPDH). **(P)** The levels of HBV core protein (HBc) in liver tissues were measured by Western blotting. β-actin served as a loading control.

Based on the results from the dose finding studies, the antiviral and immune modulatory activities of diABZI were evaluated in AAV-HBV transduced mice with an experimental strategy illustrated in Fig 3A. Briefly, AAV-HBV transduced mice were assigned into four groups at the 8th week of infection; each group had 8 or 9 mice. While group 1 received control vehicle, group 2 received low dose diABZI (0.31 mg/kg) once a week. Group 3 and group 4 received high dose diABZI (2 mg/kg) once every other week, alone or in combination with a daily dose of entecavir (ETV). At the completion of the four-week treatment, four mice from each group were euthanized on day 29. The remaining mice in group 1 or group 2 were continuingly treated with vehicle control or a reduced dose of diABZI (0.08 mg/kg) once a week for an additional 5 weeks. While diABZI treatment was terminated for both groups 3 and 4, the remaining animals in group 4 were continuingly treated with a daily dose of ETV for an additional 5 weeks.

Compared to group 1, all the treatments applied to groups 2, 3 and 4 did not change the body weight of mice during 9 weeks of experiment (Fig 3B). Serum virologic marker analysis showed that as anticipated, HBV DNA, HBsAg and HBeAg remained stable in the mice of group 1 (Fig 3C to 3E) and combination treatment of diABZI and ETV (group 4) dramatically reduced serum HBV DNA (~4.72 log reduction at day 29) (Fig 3C). However, treatment with 0.31 mg/kg (group 2) and 2 mg/kg (group 3) of diABZI resulted in similarly mean reduction of serum HBV DNA (1.05 log and 0.91 log, respectively), at day 29 (Fig 3C). All the three groups receiving diABZI treatment displayed similar extents of mean serum HBeAg decline (Fig 3E). After day 29, the remaining animals receiving 0.08 mg/kg of diABZ weekly treatment in group 2 maintained a sustained inhibition of serum HBV DNA (1.1-1.8 log) and HBeAg as compared to group 1 (Fig 3C and 3E), whereas the discontinuation of diABZI treatment in group 3 resulted in the rebound of serum HBV DNA and HBeAg to the baseline levels (Fig 3C and 3E). Interestingly, while sustained suppression of HBV DNA was observed in group 4 due to the continuation of ETV treatment, the rapid rebound of HBeAg after the termination of diABZI treatment implies that the observed reduction of HBeAg in the first 4 weeks of combination treatment is due to STING agonist-induced immune control of HBV replication (Fig 3C and 3E). No statistically significant reduction of serum HBsAg was observed in all three diABZI-treated groups (Fig 3D). However, comparative analysis of the virologic markers of individual mice in groups 1 and 2 that completed the entire nine-week therapy showed that 2/4 animals in group 2 had marked decrease of serum HBsAg (Fig 3G). HBsAb became detectable in 1/4 of mice in group 2 on day 36 and the titer of HBsAb increased to 100 mIU/ml on day 43 and remained until the end of treatment (EOT) (Fig 2I). Interestingly, the mouse that developed HBsAb response exhibited the deepest viral load reduction in response to diABZI treatment, with a 1.48-log reduction of HBV DNA, 43% reduction of HBeAg and 64% reduction of HBsAg in serum (Fig 3F, 3G and 3H). Analysis of intrahepatic HBV markers showed that on day 29, compared to vehicle-treated mice, levels of hepatic HBV DNA (Fig 3J) and pgRNA (Fig 3L) were significantly decreased in the diABZI treated group. At EOT, only one mouse in group 2 (# 5) with the highest baseline viremia showed no reduction of liver HBV DNA, as revealed by qPCR (Fig 3J) and Southern blot hybridization assays (Fig 3K), as well as pgRNA in liver (Fig 3L and 3M) and serum (Fig 3N). Western blot assay revealed that diABZI treatment did not reduce the levels of HBV core protein (HBcAg) in the liver of mice at day 29 and EOT, except for the mouse (group 2, #8) that developed HBsAb response (Fig 3P). Detection of total liver DNA using qPCR indicated that diABZI treatment had no effect on the level of the AAV vector (Fig 3O).

Histopathology analysis showed that no liver tissue injury and hepatocyte necrosis, and apoptosis were detected at EOT (S7A Fig). Immunohistochemistry (IHC) analysis showed that HBsAg-positive hepatocytes are mostly located in a zone around central vein area and diABZI treatment appeared to reduce the number of HBsAg-positive hepatocytes in this zoom (S7B Fig). In agreement with the Western blot assay results (Fig 3P), immunohistochemistry assay showed that diABZI treatment did not reduce the number of HBcAg-positive hepatocytes, except for animal # 8 in group 2 that developed HBsAb response (S7C Fig).

Taking together, our results clearly demonstrated that AAV-HBV mice are well tolerated to once a week, low dose STING agonist treatment for nine weeks. The treatment induced a persistent reduction of serum HBeAg and a significant decrease of HBV DNA in the liver and blood. Encouragingly, although the treatment did not significantly reduce the levels of serum HBsAg in most treated animals, it reduced HBsAg and induced HBsAb in 1 of 4 mice who completed the 9-week diABZI treatment.

### STING agonist treatment modulates immune response of liver microenvironment in AAV-HBV mice

**Fig 4:**
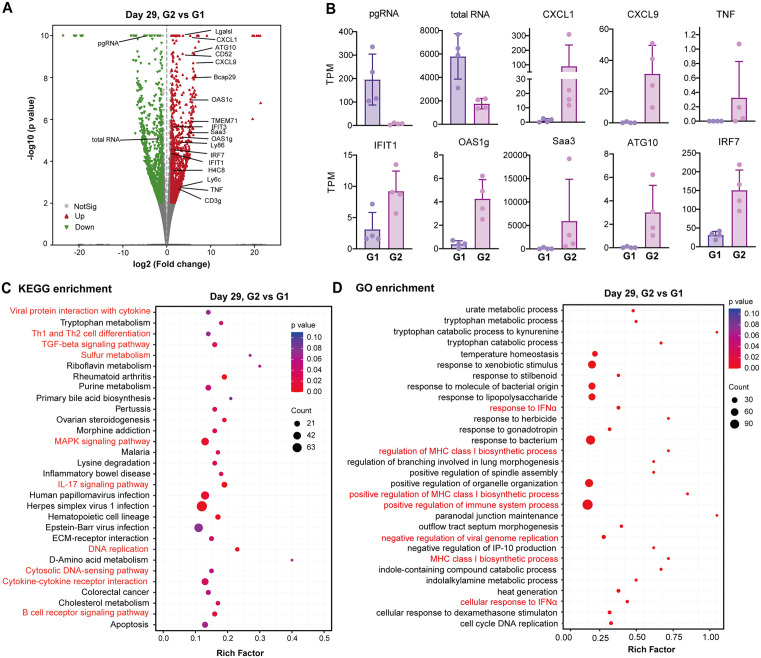
Effects of diABZI treatment on the liver expressed genes revealed by bulk RNA-sequencing analysis in rAAV-HBV transduced mice. **(A)** Volcano plot with the comparison between Group 2 and Group 1 on day 29 of treatment. Genes shown represent *p*-value≤0.05 and FDR ≤ 0.05 (upregulated genes marked in red and downregulated genes marked in green). **(B)** Histograms with transcripts per million (TPM) of the representatives with differentially expressed genes between Group 2 and Group 1. **(C-D)** KEGG or GO functional enrichment analysis with the comparison between Group 2 and Group 1 based on the differentially expressed marker genes are presented.

To assess the immune modulatory effects of STING agonist and their relationship with suppression of HBV replication in the livers of AAV-HBV mice, we first performed a bulk RNA sequencing analysis to determine the hepatic transcriptome changes induced by diABZI in AAV-HBV mice on day 29 of treatment. The results showed that the STING agonist treatment significantly altered the expression of 2092 host genes (Fig 4A), reduced the levels of intrahepatic HBV RNA (pgRNA and total RNA) and induced the expression of many ISGs (Fig 4B). Kyoto Encyclopedia of Genes and Genomes (KEGG) and Gene Ontology (GO) enrichment analyses revealed that diABZI treatment significantly altered the expression of genes involved in innate and adaptive immune responses and multiple cellular metabolism pathways (Fig 4C and 4D).

**Fig 5:**
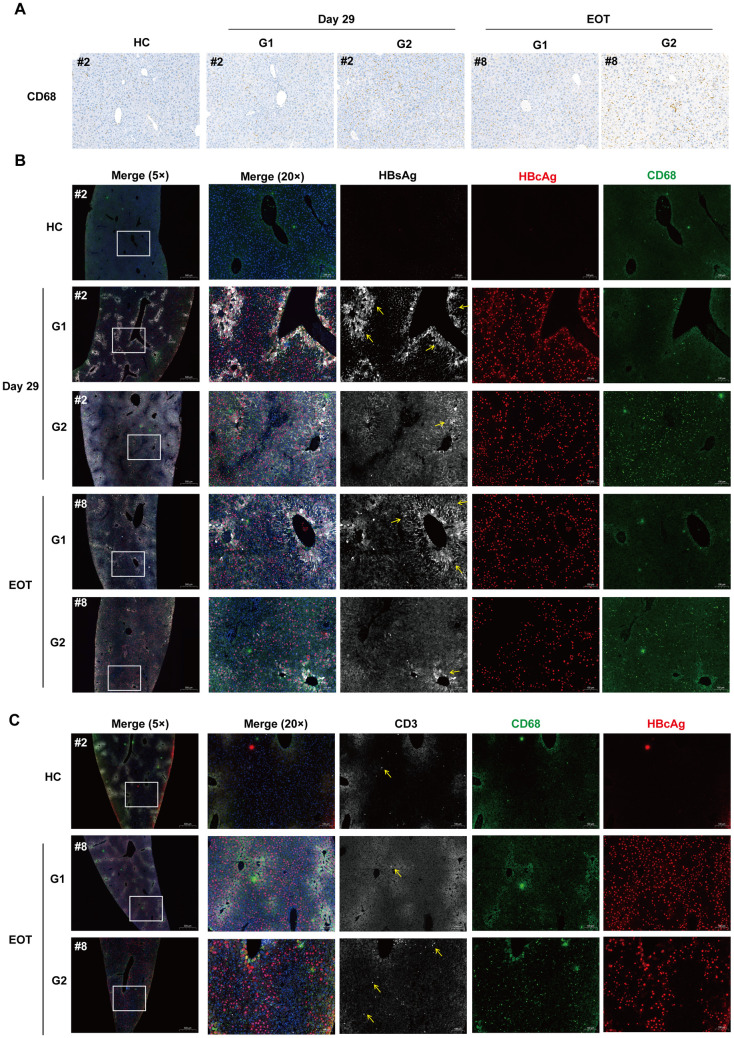
Effects of diABZI treatment on the levels of intrahepatic Kupffer cells/infiltrating monocytes (CD68^+^) and T cells (CD3^+^) in rAAV-HBV transduced mice. **(A)** The levels of Kupffer cells and infiltrating monocytes (CD68^+^) in the liver sections of representative mice from the experiment presented in Fig 3 were determined by immunohistochemical (IHC) staining. Representative images at 20 × magnification are presented. **(B)** Multicolor immunofluorescent (IF) staining of HBsAg (white, yellow arrow), HBcAg (red) and Kupffer cells and infiltrating monocytes (CD68^+^, green) in liver sections. One representative image for each group of mice from the experiment presented in Fig 3 is shown. **(C)** Multicolor IF staining of HBcAg (red), Kupffer cells and infiltrating monocytes (CD68^+^, green), and T cells (CD3^+^, white) in liver sections. One representative image for each group of mice from the experiment presented in Fig 3 is shown. HC: Healthy control.

To investigate the effects of STING agonist treatment on the composition and distribution of intrahepatic immune cells, we first performed IHC and multi-color immunofluorescent (IF) staining analysis of liver tissue sections. The results showed that diABZI treatment increased the number of CD68^+^ monocytes (Fig 5A, 5B and 5C) and CD3^+^ T lymphocytes (Fig 5C), in the livers of indicated animals on day 29 of treatment and EOT.

**Fig 6:**
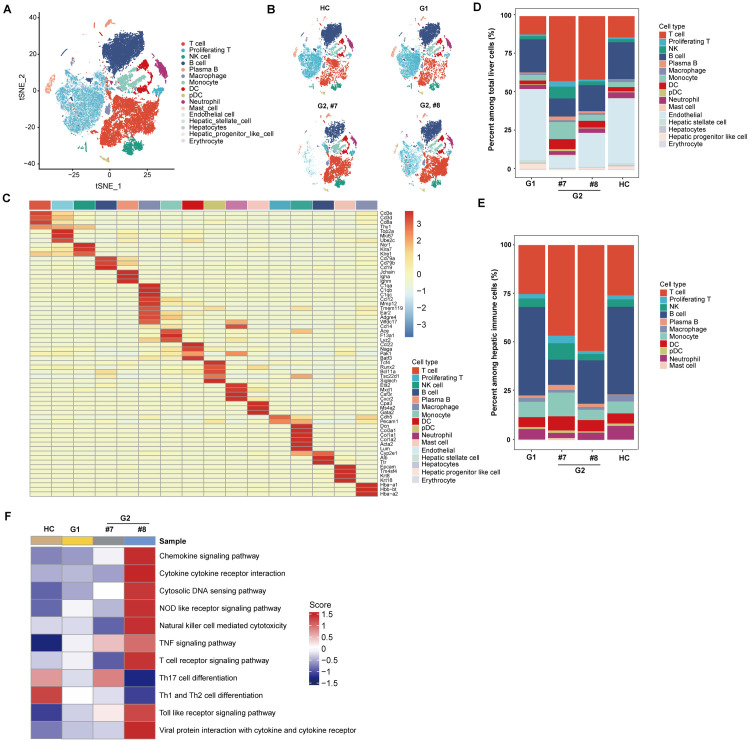
Effects of diABZI treatment on the hepatic immune cell profiles revealed by single-cell transcriptome analysis in rAAV-HBV transduced mice. Hepatic cells were collected at the end of vehicle or diABZI treatment and subjected to cell barcoding. The cDNA libraries of 5’-mRNA expression, TCR, and BCR were constructed independently, followed by high-throughput sequencing and downstream analyses. **(A)** The t-distributed stochastic neighbor embedding (t-SNE) plots of the 44337 single cells from 4 mice, including 1 healthy control mouse without rAAV-HBV transduction and diABZI treatment (HC, 11626 cells), 1 vehicle-treated AAV-HBV transduced mouse (G1, 12294 cells), 2 diABZI-treated mice (G2, #7, 9023 cells; G2, #8, 11394 cells). These cells contain 16 major clusters. **(B)** The tSNE plots for the sample-specific distribution of immune cells. **(C)** Gene expression heatmap in each cell cluster. Normalized mean expressions are shown (score). **(D)** Histogram representing the proportion of clusters among total liver cells in each sample. **(E)** Histogram representing the proportion of clusters among hepatic immune cells in each sample. **(F)** Heatmap depicting the results of Gene Set Enrichment Analysis (GSEA) using Hallmark gene sets.

To further characterize the effects of diABZI treatment on intrahepatic immune cells, single cell RNA sequencing (scRNA-seq) analysis was performed with liver cells from AAV-HBV mice treated with vehicle (HBV, #8 mouse from group 1) or diABZI (HBV + diABZI, #7 and #8 mouse from group 2). Liver cells from an age-matched healthy mouse were used as healthy control (HC). 44,337 transcriptomes of single cells with high-quality data were obtained from those four mice. After dimensionality reduction and clustering, sixteen major cell clusters were identified based on the known marker genes (Fig 6A-C). While there is no notable change in the proportion of immune cell subsets between healthy control and AAV-HBV transduced mice (Fig 6A-D), the proportion of T and B lymphocytes increased and decreased in the two AAV-HBV mice received diABZI treatment, respectively (Fig 6D and 6E). This finding is consistent with the IF staining results presented in Fig 5C. Gene Set Enrichment Analysis (GSEA) revealed that T cells in the HBsAb-producing mouse (#8) showed much stronger upregulated immune response pathways (Fig 6F), suggesting that activation of T cells may play an important role in diABZI-induced immune control of HBV replication and activation of humoral response to HBsAg.

**Fig 7:**
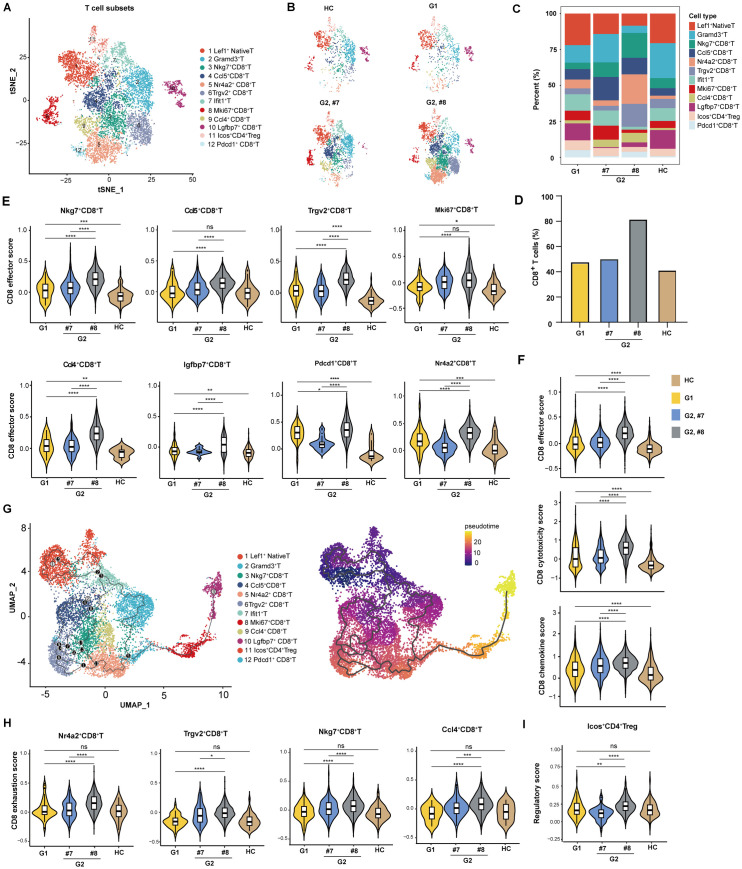
Features of hepatic T-cell subsets. **(A)** The t-SNE plots of T-cell subsets from hepatic cells of 4 mice as indicated in Fig 6. **(B)** The t-SNE plots for the treatment sample-specific distribution of T cell subsets. **(C)** Histogram representing the proportion of T cell subclusters in each sample. **(D)** Histogram representing the proportion of CD8^+^T cells among the entire T cell population in each sample. **(E)** Violin plots showing the effector scores of different CD8^+^T subclusters across different samples. **(F)** Violin plots showing the effector scores, cytotoxicity scores and chemokine scores of CD8^+^T subclusters across different samples. **(G)** RNA velocity analysis showing the transition potential among T-cell subsets. **(H)** Violin plots show the exhaustion scores of Nr4a2^+^, Trgv2^+^, Nkg7^+^ and Ccl4^+^CD8^+^T cells across different samples. **(I)** Violin plots showing the regulatory scores of Icos^+^CD4^+^Treg cells. Statistical analysis was performed by a one-sided unpaired Wilcoxon test. *P < 0.05,^**^P < 0.01,^***^P < 0.001.

To determine the modulatory effects of diABZI on T cell sub-populations in liver tissues, total intrahepatic T cells were further divided into 12 subsets (Fig 7A, 7B and 7C), according to the expression patterns of signature genes (S8A Fig). Most notably, the percentage of total CD8^+^T cells was significantly higher in the diABZI-treated AAV-HBV mouse developed HBsAb response (# 8) (Fig 7D). Moreover, both AAV-HBV transduction and diABZI treatment significantly increased the effector scores of the eight CD8^+^ T cell clusters (Fig 7E) as well as total CD8^+^T cell effector, cytotoxicity and chemokine scores (Fig 7F).

To understand the developmental transitions of T-cell clusters, RNA velocity analysis was then applied to construct the developmental trajectories of 12 T cell clusters. The liver Nr4a2^+^ CD8^+^T cell, Nkg7^+^CD8^+^T cell and Trgv2^+^CD8^+^T subsets were characterized as the same branch (Fig 7G). By examining the expression of exhaustion-specific genes (such as Tox, Nr4a1, Nr4a2, Nr4a3, Pdcd1, Tigit, Lag3, Havcr2, and CTLA4), we observed that Nr4a2^+^CD8^+^T cells have the highest exhaustion score. Nr4a2^+^CD8^+^T cells in the HBsAb-producing mouse displayed a higher activation score and effector score (Fig 7E). We also observed an increase in exhaustion score of the effector memory (Tem) CD8^+^ T subsets, including Trgv2^+^CD8^+^T, Nkg7^+^CD8^+^T and Ccl4^+^CD8^+^T cells, in diABZI-treated mice (Figs 7H and S8B). The correlation between the intrahepatic CD8^+^ T activation induced by STING stimulation and exhaustion score is consistent with a recent finding with IFN-α treatment [60]. RNA velocity analysis of T-cell clusters also revealed that Nr4a2^+^CD8^+^T cells strongly transited from central memory effector CD8^+^ T cells (Fig 7G), suggesting that modulation of the function and differentiation of CD8^+^ T cells might facilitate HBV clearance. Because CD4 expressed by Lef1^+^native T, Gramd3^+^T, and Ifit1^+^T is quite low, only one CD4^+^ subset was defined (Icos^+^CD4^+^Treg). The Icos^+^CD4^+^Treg was decreased somehow in diABZI-treated AAV-HBV-mice (Fig 7B and 7C). Notably, a higher regulatory effector score of Icos^+^CD4^+^Treg cells was observed in the HBsAb-producing mouse, indicating that the protective B-cell immune response might be related to the high level of regulatory effector function of Treg cells (Fig 7I).

The B cells were clustered into 4 subsets, including native B, memory B, plasmablasts and plasma B cells. Interestingly, the proportions of plasmablasts and plasma B cells were significantly increased in AAV-HBV mice treated with diABZI (S9 Fig).

Given that the scRNAseq experiments were done by using 1 or 2 mice per condition, future studies with larger cohorts are needed to confirm and extend our observations.

In summary, these data hint that STING agonist treatment increased the number of effector memory T cells and improved the functions of myeloid, T and B cells in AAV-HBV mice, which may contribute to its antiviral effects against HBV.

## Discussion

Chronic HBV infection is associated with the functional exhaustion of viral antigen-specific T cells [61,62] and atypical HBsAg-specific memory B cells deficient in the production of antibodies [27,63]. Because activation of STING in antigen-presenting cells and T lymphocytes may facilitate the activation of cytolytic CD8^+^ T cells and CD4^+^ T cells to facilitate the induction of humoral response against HBV, STING represents an attractive therapeutic target for the restoration of anti-HBV immunity in CHB patients [33]. DiABZI is an agonist of human and mouse STING with superior metabolic stability, tissue penetration, and activity to enhance T cell immune responses *in vivo* [53,64]. We thus used it as a tool compound to investigate the effects of STING agonist therapy on chronic HBV infection *in vivo* in the AAV-HBV mice model. The major findings of the studies and their implications are as follows.

*First*, diABZI treatment induced inflammatory cytokine responses and expression of ISGs in a broad range of doses in the livers and spleens of mice in a STING-dependent manner (Figs 1, 2, S1 and S2). RNAseq analysis also revealed that diABZI treatment of AAV-HBV mice induced extensive hepatic expression of inflammatory cytokines and ISGs (Fig 4).

*Second*, diABZI treatment promoted the activation of several distinct types of immune cells, particularly myeloid dendritic cells, M1 macrophages, and T lymphocytes in mice (S1 Fig). Intrahepatic single cell RNAseq analysis further demonstrated that diABZI treatment of AAV-HBV mice significantly expanded the numbers of intrahepatic total CD8^+^ T cell population and induced the functional activation of eight CD8^+^ T cell subtypes (Figs 6 and 7).

*Third*, the unique pharmacodynamic feature of STING agonists in mice, *i.e*., induction of inflammatory cytokines and expression of ISGs in a broad range of dosages (Fig 1), allows for flexible dosing schedule and longer treatment duration. Particularly, since the decade-long chronic HBV infection induces severe exhaustion of HBV-specific T and B cells, it is conceivable that multiple dosing and long-term STING agonist treatment is most likely required for the restoration of host antiviral immunity against HBV in CHB patients. Encouragingly, our results presented in Fig 3 clearly showed that AAV-HBV mice are well tolerated to low, but therapeutically efficacious doses of diABZI treatment for at least nine weeks.

*Fourth*, diABZI treatment significantly reduces intrahepatic pgRNA and HBV DNA replication intermediates as well as serum HBV DNA, HBeAg and pgRNA (Figs 2 and 3). These results suggest that 4 or 9 weeks of diABZI treatment primarily inhibited HBV replication, via suppression of pre-C/pgRNA transcription, but did not significantly reduce the number of HBV-replicating hepatocytes in AAV-HBV mice. This interpretation is consistent with the rebound of serum HBV DNA and HBeAg after the termination of 4-week diABZI treatment (Fig 3, treatment group 3) as well as prominent levels of HBcAg^+^ hepatocytes at EOT (Fig 5). The rapid decline of HBeAg under diABZI treatment (Fig 3E) is likely a combinatory result of transcriptional suppression of the precore RNA and post-translational regulation of pre-core protein processing and secretion. However, because core proteins are assembled into large number of empty or pgRNA-containing capsids, the persistence of intrahepatic core protein under diABZI treatment (Fig 3P) may reflect both the stability of preformed capsids and less profound suppression of pgRNA transcription. Interestingly, the mouse that developed HBsAb response at fifth week of diABZI treatment experienced the reduction of intrahepatic HBcAg and HBcAg^+^ hepatocytes at EOT, suggesting that at least some of HBV-infected hepatocytes may be cured or eliminated by STING agonist-activated HBV-specific immune responses in selected mice (Figs 3P and 5).

*Fifth*, concerning the mechanism underling diABZI suppression of HBV replication in AAV-HBV mice, the dynamics of serum and intrahepatic HBV DNA, RNA and proteins during diABZI treatment are consistent with the hypothesis that the type I IFN induced by diABZI mediates the transcription suppression of HBV pre-C/pgRNA transcription and subsequent reduction of HBV genome replication [65–67]. However, the role of STING agonist-induced activation of immune cells in the control of HBV replication in AAV-HBV mice remains to be determined. Interestingly, a recent report showed that fifteen weeks of PEG-IFN-α2 treatment of AAV-HBV transduced humanized type I IFN receptor mice not only suppress HBV replication but also induced the activation of intrahepatic monocytes and effector memory CD8^+^T cells [60]. A single-cell RNAseq analysis of peripheral immune cells in CHB patients also revealed that PEG-IFN-α treatment increased ratios of long-lived naive/memory T cells and enhanced the cytotoxicity of effector T cell [68]. These findings imply that in addition to directly suppressing HBV replication in hepatocytes [66,67,69], STING agonist-induced type I IFN response may also play important roles in modulating the function of innate and adaptive immune cells and indirectly control HBV replication in hepatocytes.

*Finally*, we noticed that the mouse producing HBsAb after diABZI treatment has the lowest baseline levels of HBV DNA, HBsAg and HBeAg in serum (Fig 3F-3I). In fact, recent clinical studies suggest that the baseline level of HBsAg is an indicator of the strength of host antiviral immune responses against HBV [65]. For instance, Kim and colleagues showed that chronic HBV carriers with higher levels of serum HBsAg (> 5,000 IU/ml) tended to have significantly higher expression of inhibitory PD-1 on CD4^+^ T cells and higher FcRL5 expression on B cells. On the contrary, checkpoint blockade with antibodies against PD-1 improved HBV-specific CD4^+^ T cell function only in patients with lower levels of serum HBsAg (< 500 IU/ml) [70]. Moreover, extensive clinical studies demonstrated that Peg-IFN-α therapy achieves a significantly higher rate (approximately 30%) of a functional cure in patients with a lower baseline level of serum HBsAg [65]. Those findings suggest that combination therapy of STING agonists with drugs that can reduce circulating HBsAg and/or other immune modulators, such as checkpoint blockade, may improve the therapeutic efficacy.

In conclusion, the work reported herein advances our understanding of STING agonist immunopharmacology and establishes a scientific basis for further development of STING agonists as immunotherapeutics for CHB and other chronic viral infections.

## Materials and methods

### Ethics statement

All animal experiments were carried out in strict accordance with the guidelines of the Animal Care and Welfare Committee in the Institute of Medicinal Biotechnology, Chinese Academy of Medical Sciences and Peking Union Medical College for the Ethics of Animal Care and Treatment (approval number: IMB-20220315-D_11_-01).

### Animal experiments

C57BL/6J male mice were purchased from SiPeiFu Biotechnology Co., Ltd (Beijing, China). STING deficient (STING-KO) mice and STING humanized (hSTING) mice were obtained from Shanghai Model Organisms Center, Inc. Mice were housed in a BSL-2 animal facility under specific pathogen-free conditions. To establish an AAV-HBV mouse model, six-week-old male mice were injected with a recombinant AAV (Adeno Associated Virus) serotype 8 (rAAV8) virus carrying 1.3 copies of the HBV genome (genotype D/serotype ayw) obtained from Beijing FivePlus Molecular Medicine Institute Co. Ltd., China in 200 μL of phosphate-buffered saline (PBS) into the tail veins as described previously [71]. Mice were also injected with PBS in the health control group. After 6 weeks or longer, AAV-HBV transduced mice were grouped based on serum HBV DNA and hepatitis B surface antigen (HBsAg) levels and treated with vehicle or indicated compounds. Blood samples were collected at the indicated time points as shown in the experimental flowchart to monitor the level of HBV DNA, HBsAg, HBeAg and HBsAb in the serum. Body weight and clinical observations were recorded daily or weekly.

### Drugs, chemicals and antibodies

Diamidobenzimidazole (diABZI) (cat no. S8796) and DMXAA (cat no. S1537) were purchased from Selleck. Entecavir (ETV) was purchased from Beijing Ouhe Technology Co., Ltd. GS-9620 was purchased from MedChemExpress. MSA-2 (cat no. DC39031) was purchased from DC chemicals. For the *in vitro* experiments, these compounds were dissolved in DMSO. For the *in vivo* experiments, diABZI was dissolved in saline (0.9% NaCl) with 1% DMSO (Sigma) and 40% PEG400 (Aladdin). DMXAA is dissolved in saline containing 5% sodium bicarbonate. Auxiliary reagents used in flow cytometry included PMA (Solarbio), lonomycin (Sigma), Brefeldin A (Solarbio), GolgiStop Protein Transport Inhibitor (BD) and Fix/Perm Buffer (BioLegend). Antibodies used have been provided in S1 Table.

### Single-cell suspension and flow cytometry analysis

Spleen was transferred into MACS gentle dissociator C tubes (Miltenyi Biotec), which contained the enzyme mix containing 2.4 mL 1 × buffer S, 50 μL enzyme D and 15 μL enzyme A from the spleen dissociation kit (Miltenyi Biotec). Then attach the C tubes upside down onto the sleeve of the gentleMACS Dissociator (Miltenyi Biotec) and run program 37C_m_SDK_1. After completion of the dissociation program, the digested cell mixtures were filtered through 70 μm MACS SmartStrainers (Miltenyi Biotec) to collect single-cell suspensions. After centrifugation, the single-cell suspensions were treated with red blood cell lysis buffer (BD) to lyse the erythrocytes. Then the single cells were suspended in MACS buffer, blocked with mouse CD16/32 antibody (BioLegend) and incubated with fluorescently labelled specific antibodies (S1 Table). Flow cytometry data were acquired and analyzed using Beckman Coulter CytoFLEX.

### Measurement of HBV DNA in serum and liver

Serum HBV DNA levels were measured using a quantitative polymerase chain (qPCR) assay kit (Sansure Biotech Inc, Changsha, Hunan, China) according to the manufacturer’s instruction. Intrahepatic HBV core DNA was extracted using 1 × lysis solution (10 mM Tris-HCl pH 8.0, 1mM EDTA, 0.5% NP40) and quantified with a qPCR assay using TransStart Tip Green qPCR SuperMix (TransGen Biotech, Beijing, China) using the ABI 7500 Fast Real-Time PCR system (Applied Biosystems) as previously described [72]. The applied primers used are shown in S2 Table. The intrahepatic HBV DNA replicative intermediates were detected by Southern blotting as previously described. HBV DNA was hybridized with a digoxigenin-labeled HBV probe synthesized with a DIG probe synthesis kit (Roche, Mannheim, Germany). The hybridization signal was detected with Chemidoc MP chemiluminescence imaging system (Bio-Rad, Hercules, CA, USA).

### Measurement of AAV vector DNA level in liver

DNA from the liver tissue was isolated using a FastPure Blood/Cell/Tissue/Bacteria DNA Isolation Mini Kit (Vazyme Biotech, Nanjing, Jiangsu, China). The level of AAV vector DNA was determined with the AceQ Universal U^+^ Probe Master Mix V2 (Vazyme Biotech) using the ABI 7500 Fast Real-Time PCR system (Applied Biosystems). Since the inverted terminal repeat (ITR) of the AAV-HBV virus is from AAV2, primers for the AAV2 ITR from the literature were used to detect the levels of the AAV vector [73]. Murine GAPDH served as an endogenous control.

### Enzyme linked immunosorbent assay (ELISA)

The levels of HBeAg, HBsAg and HBsAb in the mouse serum were quantified by commercial ELISA kits (Chemclin, Shanghai, China) under the EnVision multilabel microplate reader (PerkinElmer). The serum level of IFN-β was analyzed by an ELISA kit (MultiSciences) according to the manufacturer’s instructions.

### RNA extraction and quantitative analyses

Total RNA was extracted from cells or liver tissues using TRIzol reagent (Invitrogen) and magnetic beads RNA extraction kit (Genfine biotech, changzhou, CO.LTD) following the manufacturer’s instructions. RNA from the serum was isolated using a FastPure Viral DNA/RNA Mini Kit (Vazyme Biotech, Nanjing, Jiangsu, China). After DNase I (Invitrogen) treatment to remove contaminating DNA, the level of HBV pgRNA was determined with the TransScript II Green One-Step qRT-PCR SuperMix (TransGen Biotech) using the ABI 7500 Fast Real-Time PCR system (Applied Biosystems). The intrahepatic HBV RNA were also detected by northerning blotting using a digoxigenin-labeled HBV DNA probe as previously described [74].

The levels of murine IFNβ, IFNα, TNFα, IL-6, IFIT1, IFITM3, ISG15, Mx1, Mx2, OAS1, OAS3, STAT1, and CXCL10 were also determined by qRT-PCR assay. Murine GAPDH served as an endogenous control. The applied primers are provided in S2 Table.

### Western blot (WB) assay

The proteins were extracted from cells or liver tissues using M-PER Mammalian Protein Extraction Reagent (Thermo Fisher Scientific) with halt protease inhibitor single-use cocktail. Immunoblotting for β-actin, phosphorylated STING, p-TBK1, TBK1, p-IRF3, IRF3, p-p65, and p65 was performed with specific antibodies obtained from Cell Signaling Technology, Danvers, MA, USA (S1 Table). A rabbit polyclonal antibody against HBcAg (made by GenScript, Nanjing, Jiangsu, China) was used for detection of HBc in liver tissue lysates. The signal was detected with Omni-ECL Femto Light Chemiluminescence Kit (EpiZyme, Shanghai, China) by using Chemidoc MP chemiluminescence imaging system (Bio-Rad, Hercules, CA, USA).

### Histopathology, immunohistochemistry (IHC) and immunofluorescent (IF) staining

For histopathology, formalin-fixed, paraffin-embedded liver sections were H&E stained (Soonbio, Beijing, China). For IHC and IF, formalin-fixed and paraffin-embedded tissues were sectioned at a thickness of 3 μm. The sections were deparaffinized with xylene and rehydrated in ethanol, then placed into 3% hydrogen peroxide to block endogenous peroxidase activity. Subsequently, the liver sections were incubated with primary antibodies at 4°C overnight and then incubated with species-specific secondary antibodies at room temperature (RT). The antibodies used are shown in S1 Table. Images were acquired using a 3D Histech MIDI pannoramic scanner (3D Histech, Hungary).

### Bulk liver RNA sequencing and data analysis

Total liver RNA was extracted using TRIzol reagent (Invitrogen). RNA integrity was accurately detected using the Agilent 4200 system. RNA degradation and contamination were detected by electrophoresis on 1% agarose gel. RNA sequencing library was built using the ALFA-SEQ RNA Library Prep Kit following the manufacturer’s recommendations. Then the library was sequenced on the Illumina PE150 platform by Guangdong Meg Gene Biotechnology Co., LTD. (Guangzhou, China). After quality control and reference genome alignment, quantitative analysis of gene expression levels and differential expression analysis were performed. The RSEM (version 1.3.3) https://github.com/deweylab/RSEM was used for each gene read counts. To make the expression levels of genes comparable between different genes and different experiments, the TPM (transcripts per million) of each gene was calculated. PCA (principal components analysis) were used to reveal the relationship between all samples. DESeq2 (v1.34.0) http://www.bioconductor.org/packages/release/bioc/html/ DESeq2.html was used to analyze two conditions/groups of differentially expressed genes. FDR (false discovery rate) ≤0.05 and | log2 (fold change) | ≥ 1 was considered as differentially expressed genes. These genes were then enriched for GO (Gene Ontology, http://www.geneontology.org) and KEGG (said 49-year-old kyoko Encyclopedia of Genes and Genomes, http://www.genome.jp/kegg/) functions using the “clusterProfler (v4.2.2)”, respectively. GO terms and KEGG pathways with FDR ≤ 0.05 were screened for significant enrichment.

### Single-cell RNA sequencing and data analysis

All the RNA extraction, library preparation and sequencing were done by the Beijing SeekGene BioSciences Co., Ltd (SeekGene). Please find the details below. After resection, fresh liver tissues were washed in ice-cold PBS and then dissociated using Multi-tissue dissociation kit 2 (Miltenyi) as instructions. After removal of erythrocytes, debris and dead cells, cell count, and viability were estimated using a fluorescence Cell Analyzer (Countstar Rigel S2) with an AO/PI reagent. Single-cell RNA-Seq libraries were performed using SeekOne Digital Droplet Single Cell 5’ library preparation kit (SeekGene). The resulting amplified cDNA was sufficient to construct 5’ gene expression libraries and V(D)J enriched libraries. The indexed sequencing libraries were cleaned up with SPRI beads, quantified by quantitative PCR (KAPA Biosystems KK4824) and then sequenced on illumina NovaSeq 6000 with PE150 read length. After quality control and filtering, library-size normalization to each cell was performed by NormalizeData of Seurat (version 4.0.0). FindAllMarkers was used to compare each cluster to all others to identify cluster-specific marker genes. The clustering differential expressed genes were considered significant if the adjusted P-value was less than 0.05 and the avg_log2FC was ≥ 0. Seurat Function AddModuleScore was used to combine the expression of gene list. Then, we performed PCA and dimension reduction techniques to visualize cell distance in reduced two-dimensional space, including Uniform Manifold Approximation and Projection (UMAP) and t-distributed Stochastic Neighbor Embedding (t-SNE). The FindMarkers function was used to identify differentially expressed genes (DEGs) across or between distinct groups. Gene Ontology (GO) enrichment analysis of DEGs was implemented by the clusterProfiler R package. The gene set enrichment analysis (GSEA) was performed using the Molecular Signatures Database. Monocle 3 package was used to determine the potential lineage differentiation. RNA velocity estimation was implemented using the scVelo python package (v0.2.5).

### Statistical analysis

Results were expressed as mean ± standard error of the mean (SD) and analyzed with unpaired two-tailed Student’s *t*-test (comparisons between two groups) or One-way ANOVA or Two-way ANOVA with Holm-Sidak multiple comparisons test. A threshold of *P* < 0.05 was defined as statistically significant. Statistical analysis and graphic representations were performed using GraphPad Prism software.

## Supporting information

S1 Fig diABZI treatment alters the distribution and function of mouse splenocytes. C57BL/6J male mice were treated with 0.31 mg/kg of diABZI or vehicle by intraperitoneal injection. At 16 h after treatment, the immune cells in the spleen were analyzed by flow cytometry (n = 4 or 5 per group). (A) Quantification of myeloid cells including monocyte, neutrophil macrophage, M1-like macrophage, M2-like macrophage, DC, mature DC (CD80^+^), and pDC. (B) Quantification of CD4^+^ T cells, activated CD4^+^ T (CD25^+^ or CD69^+^), Tfh CD4^+^T, Th1, Th2, Th17, and Treg CD4^+^T cells. (C) Quantification of CD8^+^ T cells, activated CD8^+^ T (CD69^+^), IFNγ^+^ CD8^+^T, and Granzyme-B^+^ CD8^+^T cells. (D) Quantification of NK cells, IFNγ^+^ NK, Granzyme-B^+^ NK, NKT, IFNγ^+^ NKT, Granzyme-B^+^ NKT cells. (E) Quantification of TCRγ/δT cells. (F) Splenocytes isolated from C57BL/6J male mice were mock-treated (control) or treated with diABZI (0.5 μM) or DMXAA (50 μM) for 3 h. Phosphorylated STING, p-TBK1, TBK1, p-IRF3, IRF3, p-p65, and p65 in cell lysates were detected by Western blot assays with specific antibodies. β-actin served as a loading control. ^*^, ^**^ and ^***^ indicates *P* < 0.05, 0.01 and 0.001, respectively. ns indicates no significant difference.
(TIF)

S2 Fig Effect of diABZI treatment on HBV replication in rAAV-HBV transduced STING knockout (KO) mice. (A) Validation of STING KO mice by detecting the STING protein level in the spleen by Western blotting assay. (B) Effect of STING agonists DMXAA (50 μM), diABZI (0.5 μM) and MSA-2 (15 μM) on the expression of CXCL10, IL-6, IFIT1, ISG15, Mx1 and Mx2 in splenocytes from STING KO or WT mice. Data are expressed as fold induction of gene expression relative to that in negative control (NC) treated with DMSO. (C) Experimental setup for *in vivo* efficacy study in STING knockout mice. All mice received a single i.v. injection of 8 × 10^10^ vg of rAAV8-HBV1.3. On week 11 after inoculation, mice were grouped and treated with 0.08 mg/kg of diABZI or vehicle. Images of the mouse, syringe, and liver were sourced from https://openclipart.org/17558, 282069, and 37315, respectively. (D-F) Effect of diABZI on the serum levels of HBV DNA, HBsAg and HBeAg in rAAV-HBV transduced STING KO mice. (G-H) Effect of diABZI on the intrahepatic HBV DNA and pgRNA were determined by qPCR assay and plotted as log copies per 1 µg of total DNA and fold of change related to that in rAAV-HBV transduced STING KO mice received vehicle treatment.
(TIF)

S3 Fig The body weight of mice during diABZI treatment. 6-week-old male C57BL/6J mice were treated with diABZI (0.625, 1.25 and 2.5 mg/kg) or vehicle by either intraperitoneal (IP) injection (A) or tail intravenous (IV) injection (B) on day 0 and day 7 (n = 5 per group). Mean values ± SD are plotted for each group.
(TIF)

S4 Fig Tolerability of mice to STING agonist treatment. (A) 12-week-old male C57BL/6J mice were treated with 0.31 mg/kg of diABZI, 20 mg/kg of DMXAA or vehicle by IP injection at the indicated times (n = 4–5/group). The body weight of mice was monitored at the beginning of treatment and during a 20-day dosing period. Mean values ± SD are plotted for each group. (B) 12-week-old male C57BL/6J mice were treated with diABZI (0.08, 0.16 and 0.31 mg/kg), DMXAA (10 and 15 mg/kg) or vehicle by IP injection at the indicated times (N: n = 2; the others, n = 4–5/group). The body weight of mice was monitored at the beginning of treatment and during a 72-day dosing period. Mean values ± SD are plotted for each group. (C) Hematoxylin and eosin (H&E) staining of liver tissue from each group in (B) for analyzing the histological changes. Representative images at 20×magnification are presented. (D-E) The serum ALT and Cre levels at the end of the treatment are determined. Mean values ± SD are plotted for each group.
(TIF)

S5 Fig Effect of diABZI and DMXAA on the innate immune response in mice at lower doses. C57BL/6J male mice were treated with diABZI (0.04, 0.08, 0.16 and 0.31 mg/kg), DMXAA (10 and 15 mg/kg) or vehicle by IP injection. At 2 h after treatment, the mRNA levels of IFIT1 (A), ISG15 (B), Mx1 (C), OAS3 (D), CXCL10 (E) and STAT1 (F) in the livers were determined by qRT-PCR (normalized to GAPDH) (n = 4/group). Data (mean values ± standard deviations) are expressed as fold induction of gene expression relative to that in vehicle-treated control.
(TIF)

S6 Fig Antiviral activity of diABZI in a HBV hydrodynamic mouse model. (A) Two days after hydrodynamic injection of 10 μg of HBV 1.3mer plasmid (day 0), six-week-old male C57BL/6 mice were treated with diABZI (0.31mg/kg) or the vehicle via IP injection every other day. Mice that did not receive the HBV1.3 plasmid injection served as the negative control. Blood samples were collected at indicated time points (one day before, and 1, 3, and 5 days after treatment). Images of the mouse, syringe, and liver were sourced from >https://openclipart.org/17558, 282069, and 37315, respectively. (B-E) Serum HBV DNA was quantified by qPCR. (F) Liver tissues on day 1 were lysed in 1 × lysis solution (10 mM Tris-HCl pH8.0, 1 mM EDTA, 1% NP40). After DNase I treatment, HBV core DNA was extracted and then determined by qPCR (normalized per 1 ng of DNA). Mean values ± SD are plotted for each group. ^***^P < 0.001,^**^P < 0.01, ^*^P < 0.05 by Student’s *t*-test.
(TIF)

S7 Fig Histopathology and immunohistochemistry analysis of liver tissues. (A) Hematoxylin and eosin (H&E) staining of liver tissues from vehicle or diABZI treated rAAV-HBV transduced mice (Group 1 and Group 2) in the experiment presented in Fig 3. (B-C) The levels of HBsAg and HBcAg in the liver tissues were determined by immunohistochemistry (IHC). Representative images are presented to highlight the predominant staining patterns found in each mouse. Scale bar = 100 μm.
(TIF)

S8 Fig Features of hepatic T-cell subsets. (A) Gene expression heatmap in T cell cluster. Normalized mean expressions are shown (score). (B) The exhaustion scores of CD8^+^T cell subsets across different samples.
(TIF)

S9 Fig Immunological features of B cell subsets. (A) The t-SNE plots of B cell subsets. (B) t-SNE plots of B cell subsets showing sample distribution. (C) Violin plots showing gene expression level in each cell cluster. (D) Histogram representing the proportion of B cell subsets in each sample.
(TIF)

S1 Table Antibodies. (DOCX)

S2 Table PCR primer sequences. (DOCX)
