# Supplementary figures and images for: Antiviral and immune modulatory activities of STING agonists in a mouse model of persistent hepatitis B virus infection
Source: PLoS Pathog. 2025 Dec 9;21(12):e1013709. doi: 10.1371/journal.ppat.1013709 (PMC12700435; doi:10.1371/journal.ppat.1013709)

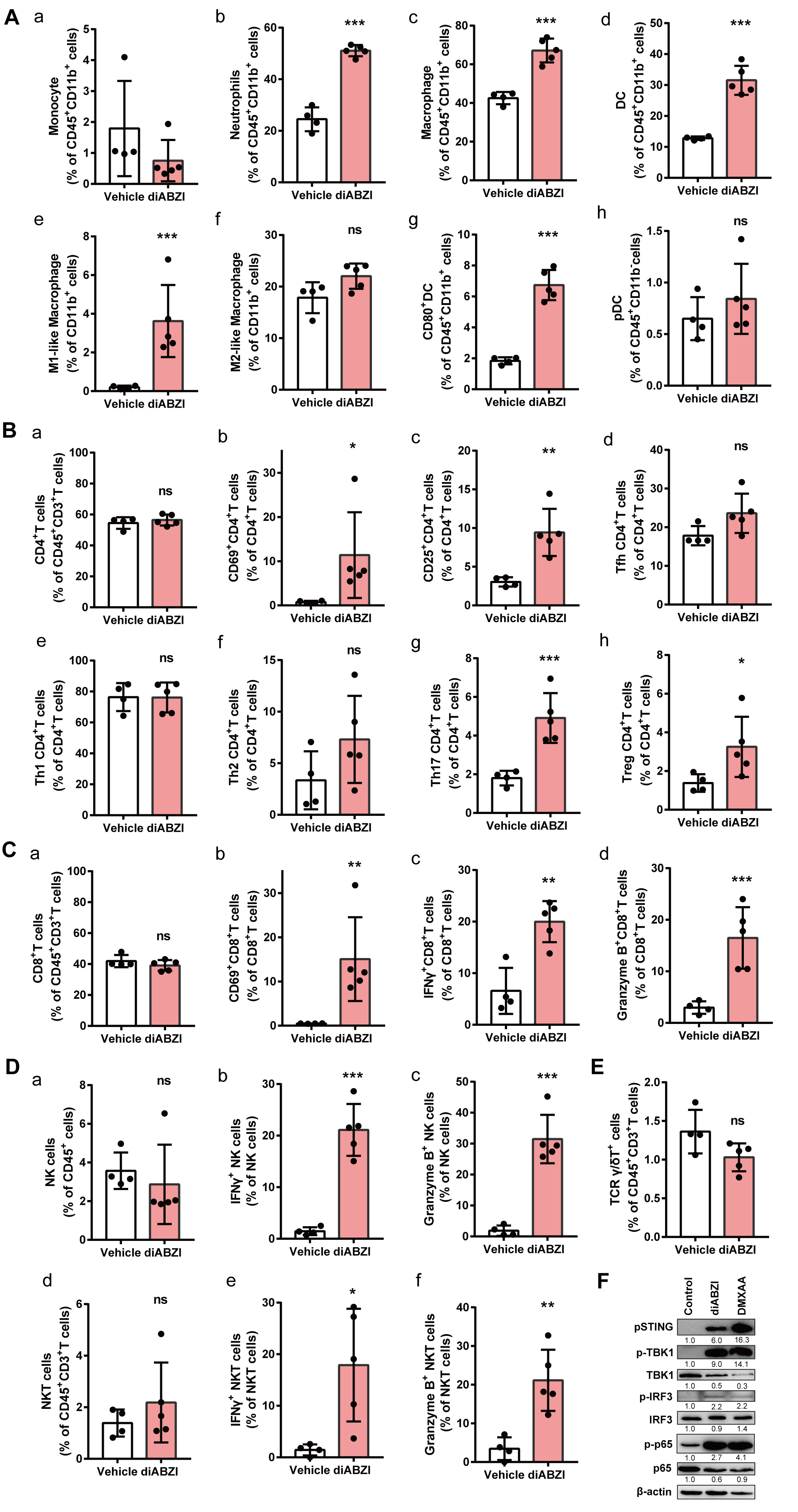

Supplement: S1 Fig — C57BL/6J male mice were treated with 0.31 mg/kg of diABZI or vehicle by intraperitoneal injection. At 16 h after treatment, the immune cells in the spleen were analyzed by flow cytometry (n = 4 or 5 per group). (A) Quantification of myeloid cells including monocyte, neutrophil macrophage, M1-like macrophage, M2-like macrophage, DC, mature DC (CD80+), and pDC. (B) Quantification of CD4+ T cells, activated CD4+ T (CD25+ or CD69+), Tfh CD4+T, Th1, Th2, Th17, and Treg CD4+T cells. (C) Quantification of CD8+ T cells, activated CD8+ T (CD69+), IFNγ+ CD8+T, and Granzyme-B+ CD8+T cells. (D) Quantification of NK cells, IFNγ+ NK, Granzyme-B+ NK, NKT, IFNγ+ NKT, Granzyme-B+ NKT cells. (E) Quantification of TCRγ/δT cells. (F) Splenocytes isolated from C57BL/6J male mice were mock-treated (control) or treated with diABZI (0.5 μM) or DMXAA (50 μM) for 3 h. Phosphorylated STING, p-TBK1, TBK1, p-IRF3, IRF3, p-p65, and p65 in cell lysates were detected by Western blot assays with specific antibodies. β-actin served as a loading control. *, ** and *** indicates P < 0.05, 0.01 and 0.001, respectively. ns indicates no significant difference. (TIF) [file ppat.1013709.s001.tif]

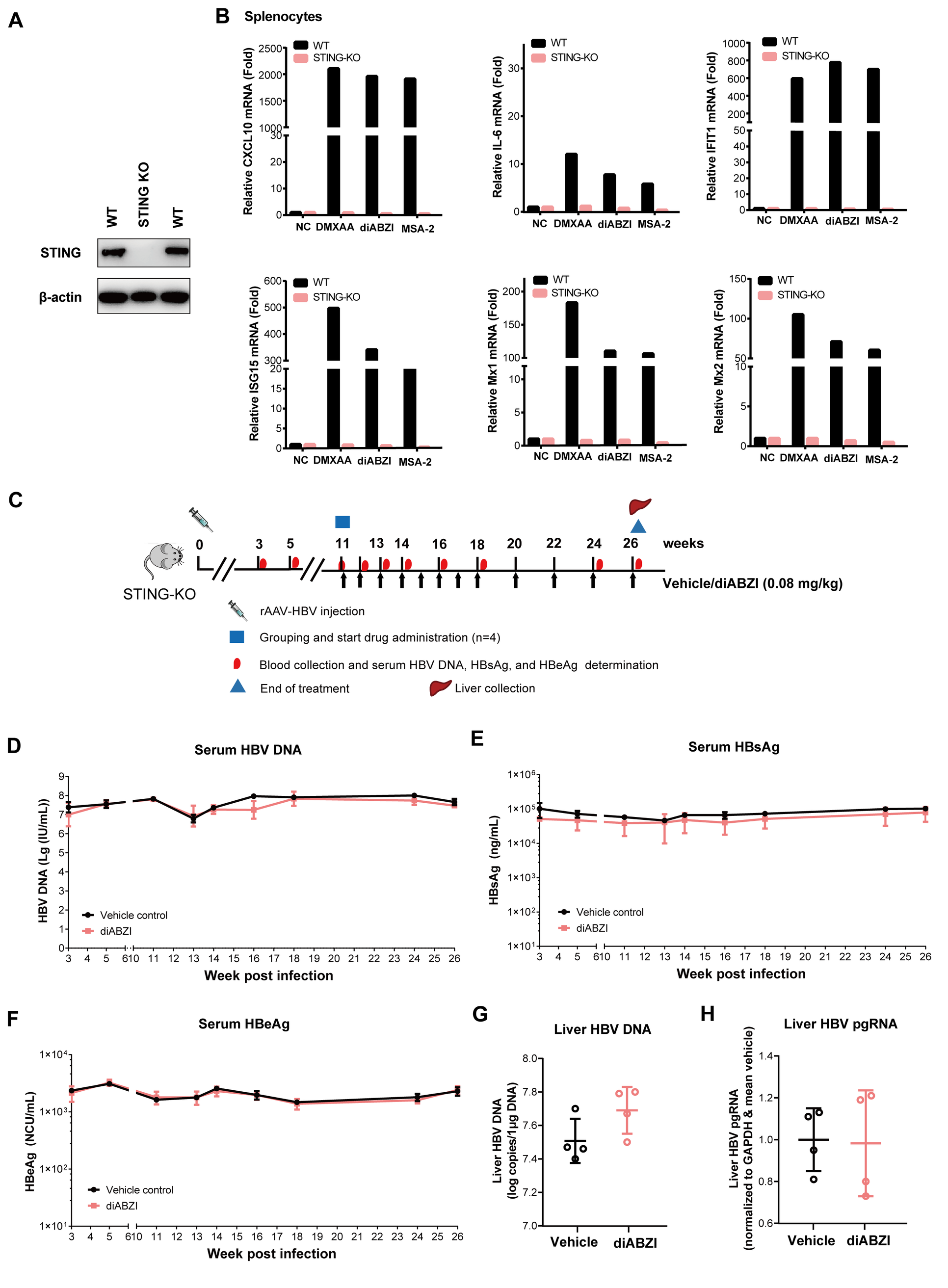

Supplement: S2 Fig — (A) Validation of STING KO mice by detecting the STING protein level in the spleen by Western blotting assay. (B) Effect of STING agonists DMXAA (50 μM), diABZI (0.5 μM) and MSA-2 (15 μM) on the expression of CXCL10, IL-6, IFIT1, ISG15, Mx1 and Mx2 in splenocytes from STING KO or WT mice. Data are expressed as fold induction of gene expression relative to that in negative control (NC) treated with DMSO. (C) Experimental setup for in vivo efficacy study in STING knockout mice. All mice received a single i.v. injection of 8 × 10^10^ vg of rAAV8-HBV1.3. On week 11 after inoculation, mice were grouped and treated with 0.08 mg/kg of diABZI or vehicle. Images of the mouse, syringe, and liver were sourced from https://openclipart.org/17558, 282069, and 37315, respectively. (D-F) Effect of diABZI on the serum levels of HBV DNA, HBsAg and HBeAg in rAAV-HBV transduced STING KO mice. (G-H) Effect of diABZI on the intrahepatic HBV DNA and pgRNA were determined by qPCR assay and plotted as log copies per 1 µg of total DNA and fold of change related to that in rAAV-HBV transduced STING KO mice received vehicle treatment. (TIF) [file ppat.1013709.s002.tif]

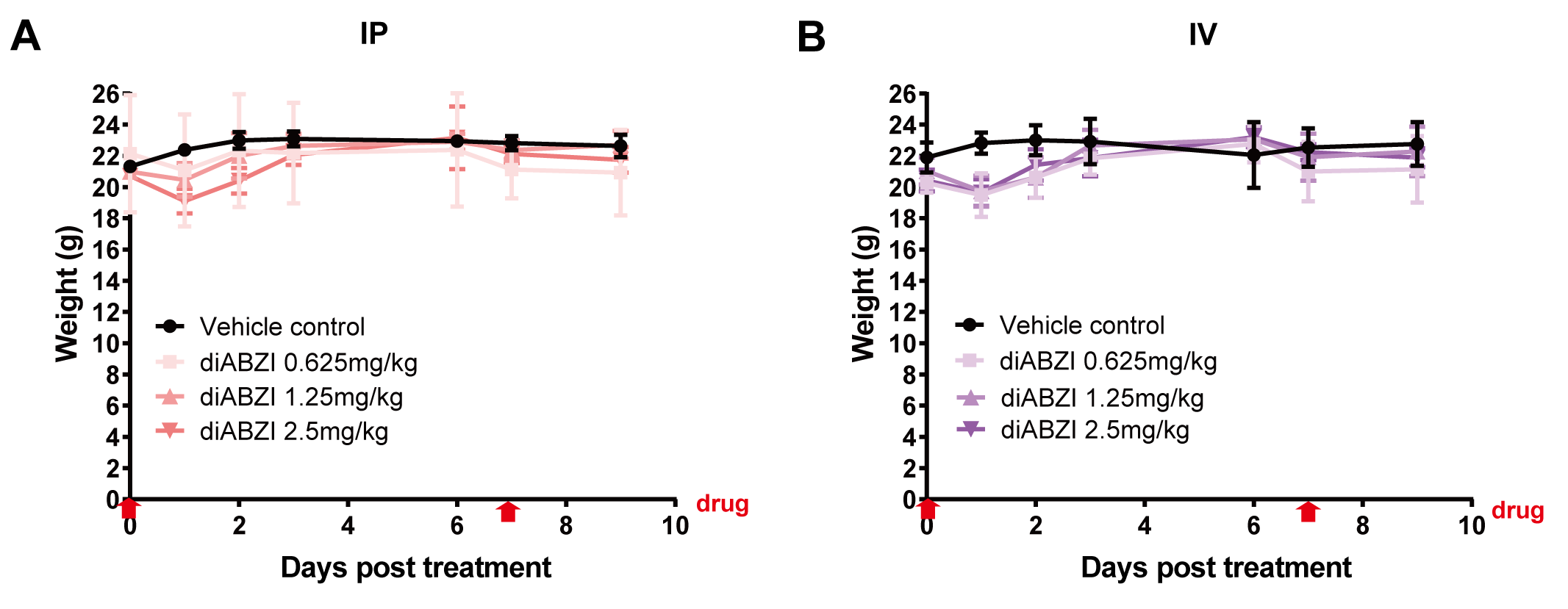

Supplement: S3 Fig — 6-week-old male C57BL/6J mice were treated with diABZI (0.625, 1.25 and 2.5 mg/kg) or vehicle by either intraperitoneal (IP) injection (A) or tail intravenous (IV) injection (B) on day 0 and day 7 (n = 5 per group). Mean values ± SD are plotted for each group. (TIF) [file ppat.1013709.s003.tif]

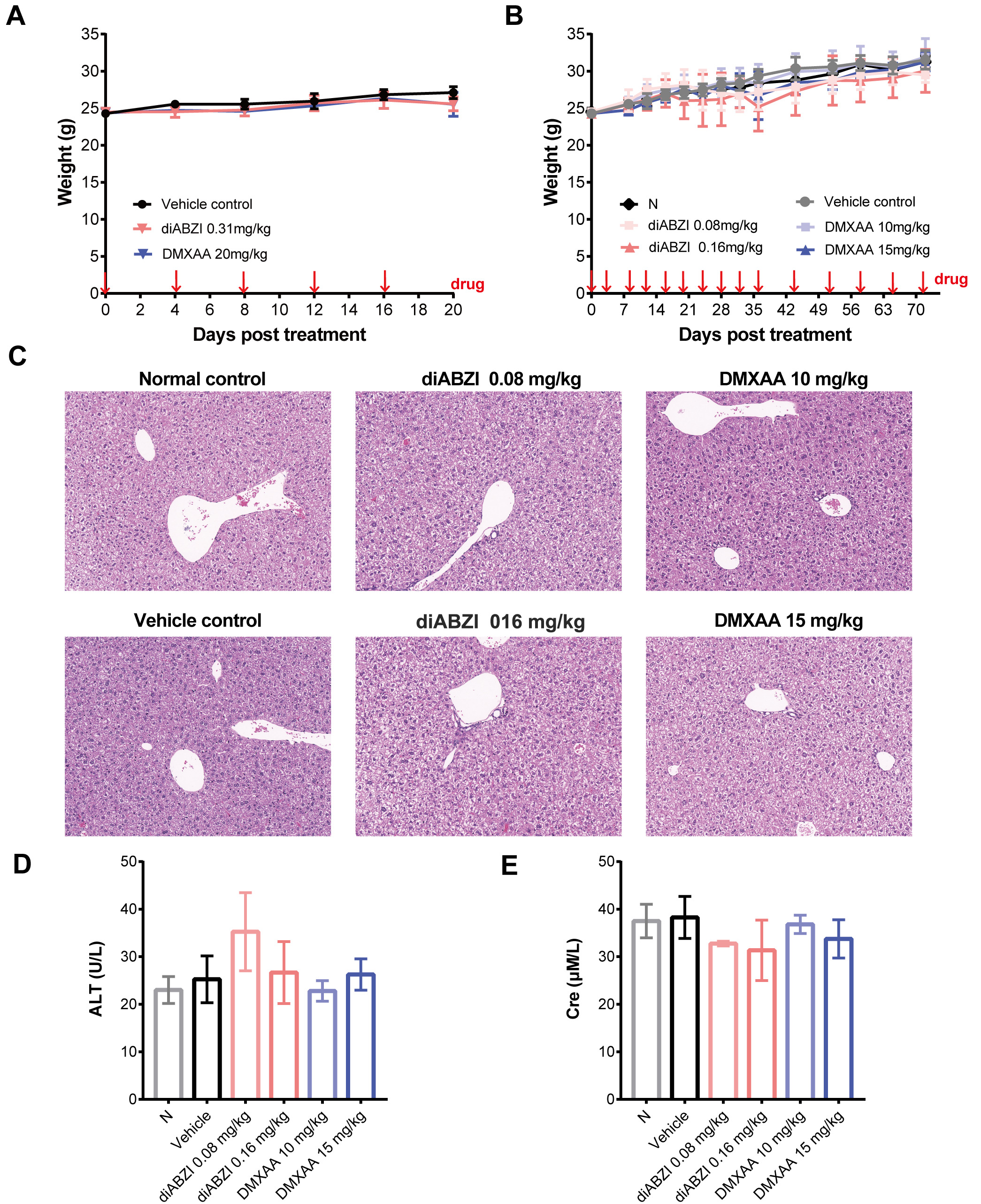

Supplement: S4 Fig — (A) 12-week-old male C57BL/6J mice were treated with 0.31 mg/kg of diABZI, 20 mg/kg of DMXAA or vehicle by IP injection at the indicated times (n = 4–5/group). The body weight of mice was monitored at the beginning of treatment and during a 20-day dosing period. Mean values ± SD are plotted for each group. (B) 12-week-old male C57BL/6J mice were treated with diABZI (0.08, 0.16 and 0.31 mg/kg), DMXAA (10 and 15 mg/kg) or vehicle by IP injection at the indicated times (N: n = 2; the others, n = 4–5/group). The body weight of mice was monitored at the beginning of treatment and during a 72-day dosing period. Mean values ± SD are plotted for each group. (C) Hematoxylin and eosin (H&E) staining of liver tissue from each group in (B) for analyzing the histological changes. Representative images at 20×magnification are presented. (D-E) The serum ALT and Cre levels at the end of the treatment are determined. Mean values ± SD are plotted for each group. (TIF) [file ppat.1013709.s004.tif]

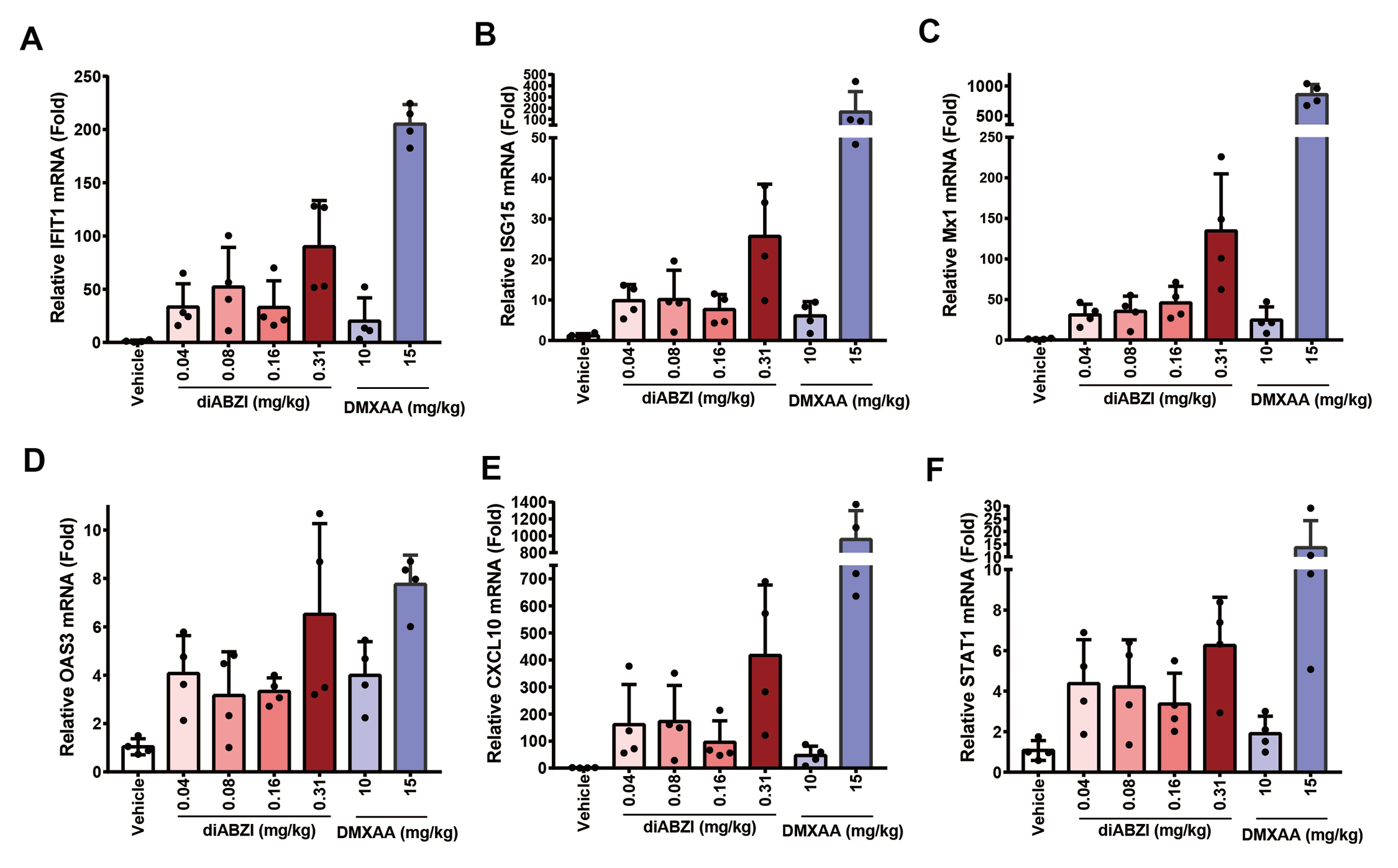

Supplement: S5 Fig — C57BL/6J male mice were treated with diABZI (0.04, 0.08, 0.16 and 0.31 mg/kg), DMXAA (10 and 15 mg/kg) or vehicle by IP injection. At 2 h after treatment, the mRNA levels of IFIT1 (A), ISG15 (B), Mx1 (C), OAS3 (D), CXCL10 (E) and STAT1 (F) in the livers were determined by qRT-PCR (normalized to GAPDH) (n = 4/group). Data (mean values ± standard deviations) are expressed as fold induction of gene expression relative to that in vehicle-treated control. (TIF) [file ppat.1013709.s005.tif]

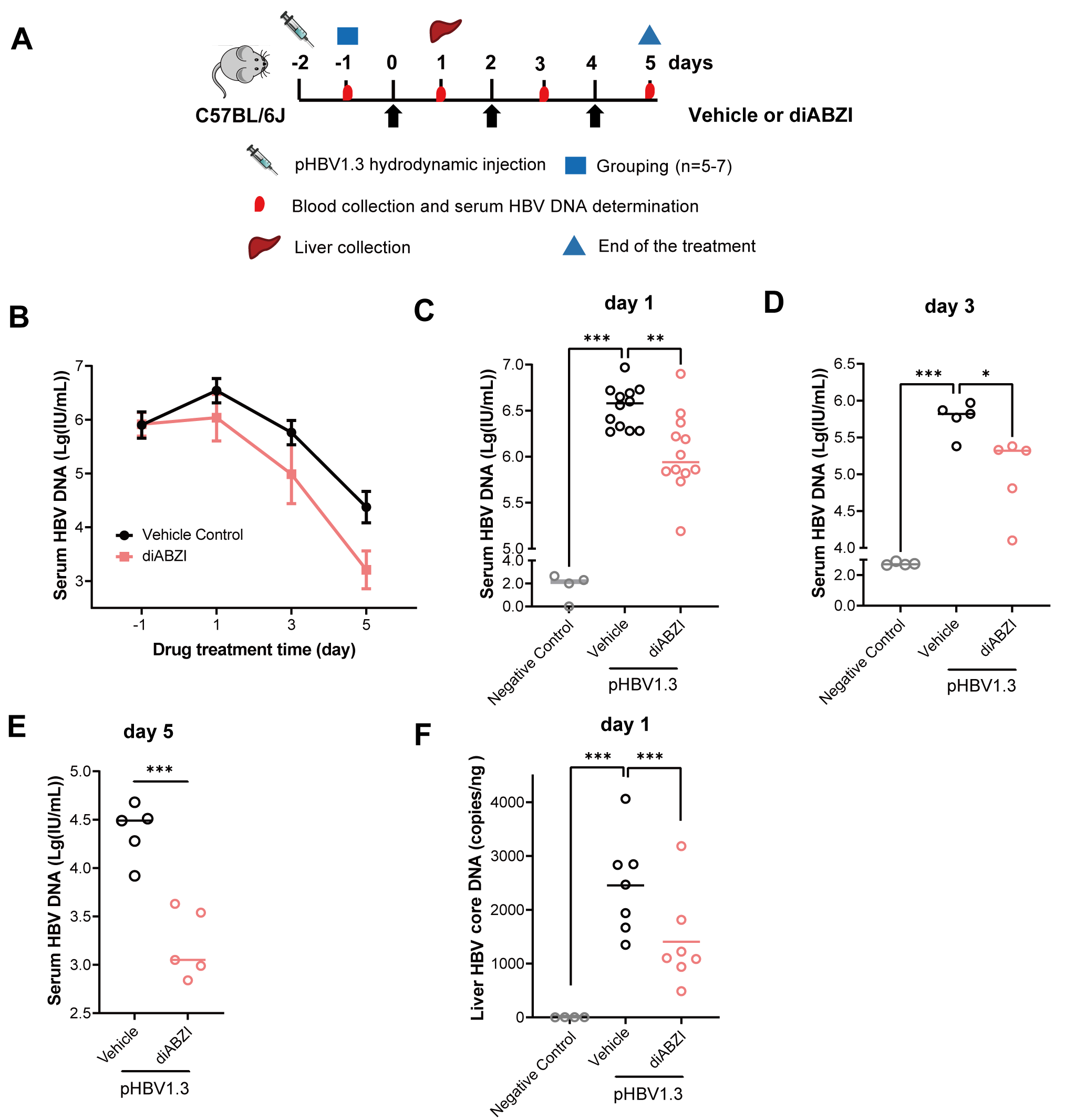

Supplement: S6 Fig — (A) Two days after hydrodynamic injection of 10 μg of HBV 1.3mer plasmid (day 0), six-week-old male C57BL/6 mice were treated with diABZI (0.31mg/kg) or the vehicle via IP injection every other day. Mice that did not receive the HBV1.3 plasmid injection served as the negative control. Blood samples were collected at indicated time points (one day before, and 1, 3, and 5 days after treatment). Images of the mouse, syringe, and liver were sourced from >https://openclipart.org/17558, 282069, and 37315, respectively. (B-E) Serum HBV DNA was quantified by qPCR. (F) Liver tissues on day 1 were lysed in 1 × lysis solution (10 mM Tris-HCl pH8.0, 1 mM EDTA, 1% NP40). After DNase I treatment, HBV core DNA was extracted and then determined by qPCR (normalized per 1 ng of DNA). Mean values ± SD are plotted for each group. ***P < 0.001,**P < 0.01, *P < 0.05 by Student’s t-test. (TIF) [file ppat.1013709.s006.tif]

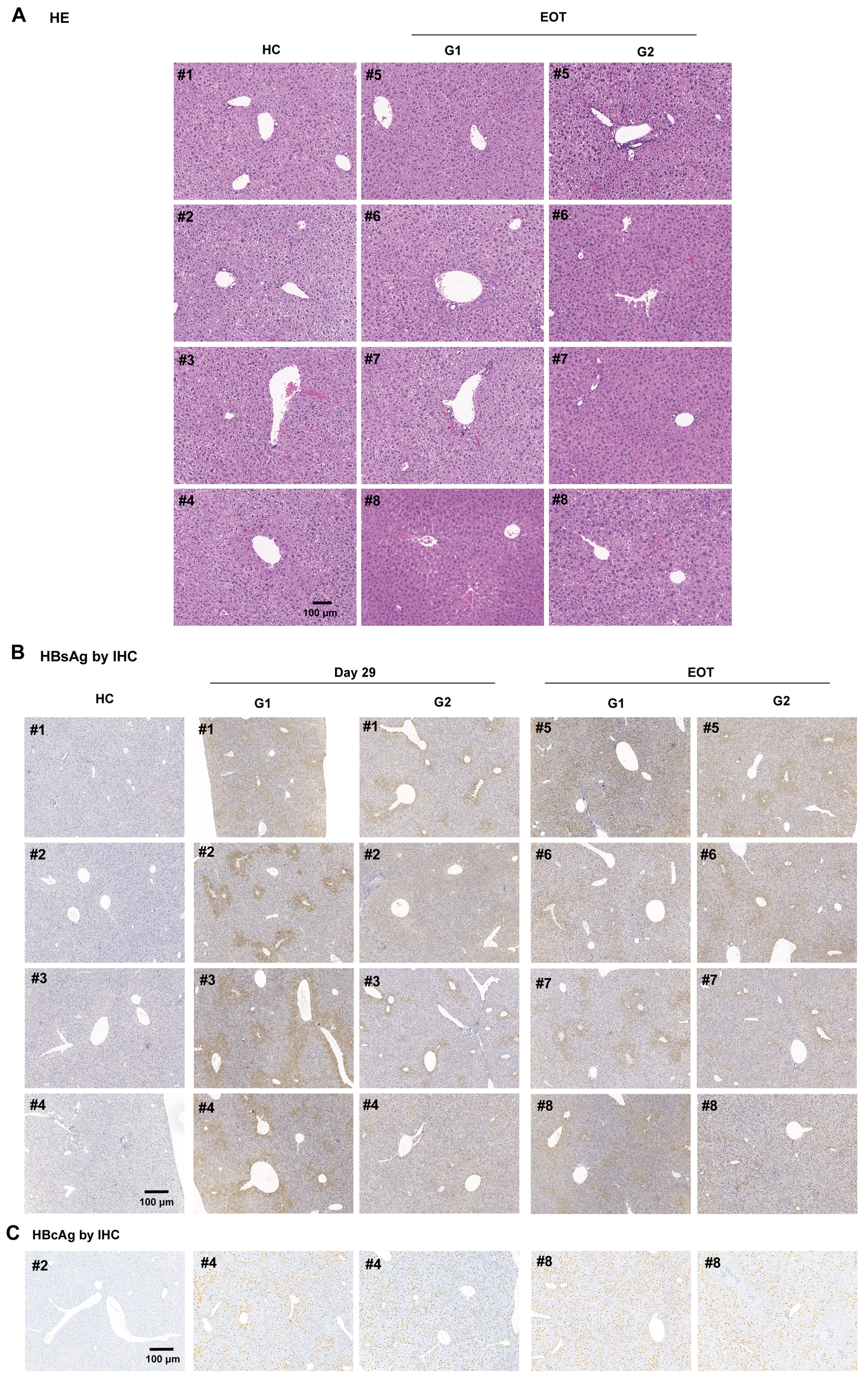

Supplement: S7 Fig — (A) Hematoxylin and eosin (H&E) staining of liver tissues from vehicle or diABZI treated rAAV-HBV transduced mice (Group 1 and Group 2) in the experiment presented in Fig 3. (B-C) The levels of HBsAg and HBcAg in the liver tissues were determined by immunohistochemistry (IHC). Representative images are presented to highlight the predominant staining patterns found in each mouse. Scale bar = 100 μm. (TIF) [file ppat.1013709.s007.tif]

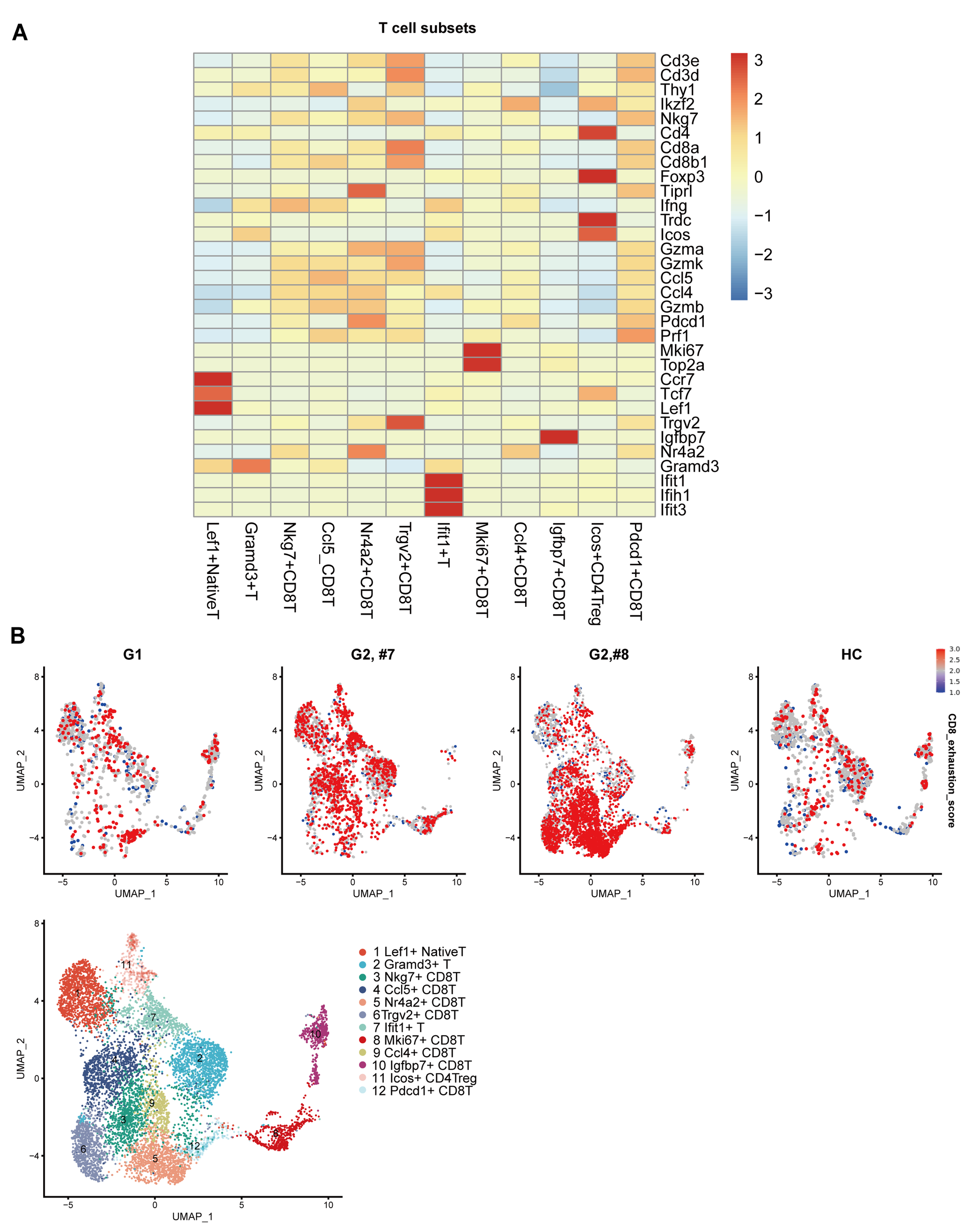

Supplement: S8 Fig — (A) Gene expression heatmap in T cell cluster. Normalized mean expressions are shown (score). (B) The exhaustion scores of CD8+T cell subsets across different samples. (TIF) [file ppat.1013709.s008.tif]

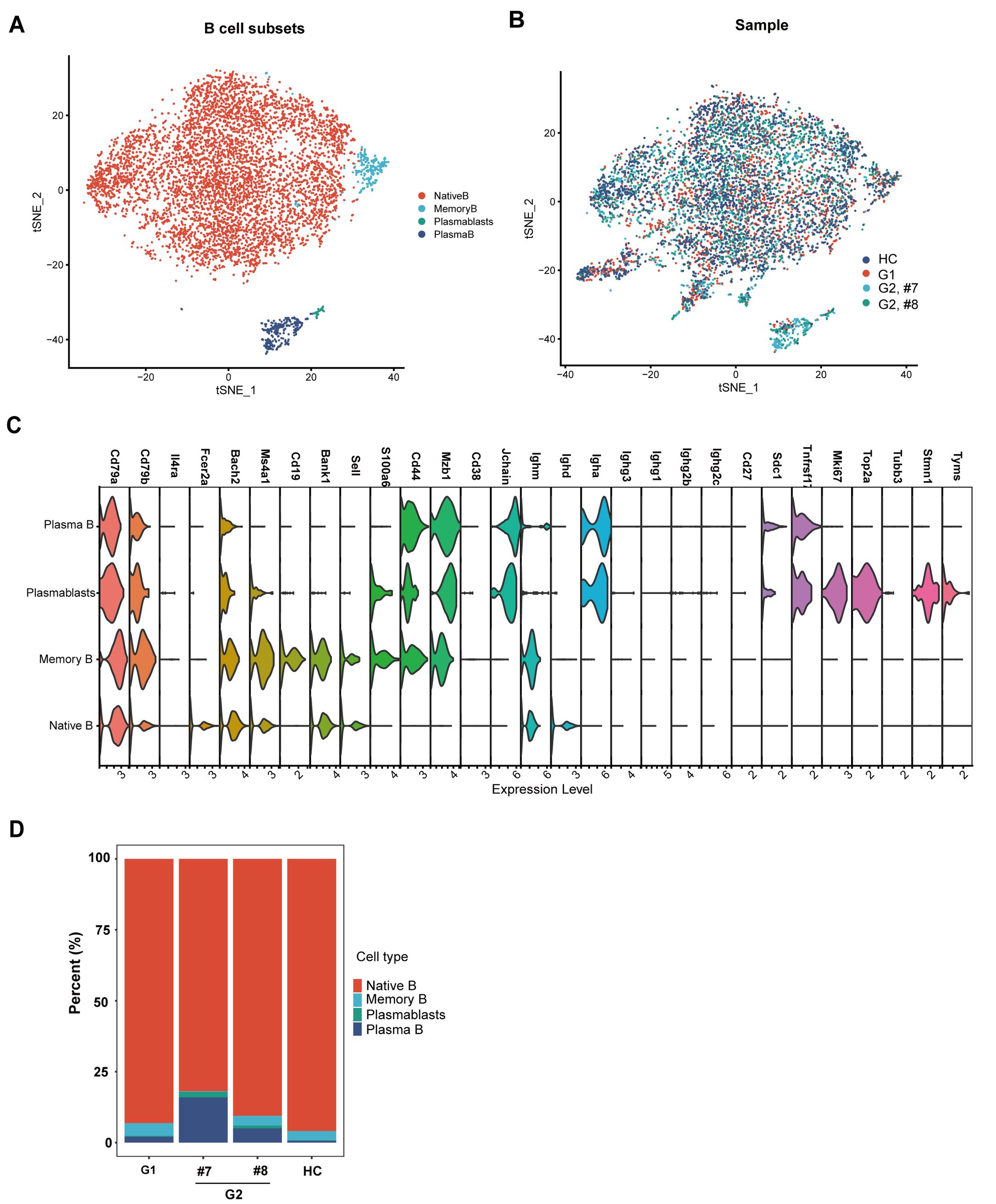

Supplement: S9 Fig — (A) The t-SNE plots of B cell subsets. (B) t-SNE plots of B cell subsets showing sample distribution. (C) Violin plots showing gene expression level in each cell cluster. (D) Histogram representing the proportion of B cell subsets in each sample. (TIF) [file ppat.1013709.s009.tif]
