# Supplementary material for: Antiviral and immune modulatory activities of STING agonists in a mouse model of persistent hepatitis B virus infection
Source: PLoS Pathog. 2025 Dec 9;21(12):e1013709. doi: 10.1371/journal.ppat.1013709 (PMC12700435; doi:10.1371/journal.ppat.1013709)
Supplement: S1 Table — (DOCX) [file ppat.1013709.s010.docx]

**Table S1. Antibodies.**

| **Name** | **Supplier** | **Cat no.** | **Experiment** |
| --- | --- | --- | --- |
| Rabbit Ab against Hepatitis B Virus Surface Antigen | GeneTex | GTX40878 | IHC, IF |
| Rabbit Hepatitis B Virus Core Antigen polyclonal Antibody | GenScript | Customized | IHC, WB, IF |
| Rabbit anti-mouse CD68 | abcam | ab125212 | IHC, IF |
| Rabbit anti-mouse CD3 | abcam | ab16669 | IHC, IF |
| APC/Cyanine7 anti-mouse CD45 Antibody | Biolegend | 103116 | Flow cytometry |
| FITC anti-mouse CD3 Antibody | Biolegend | 100204 | Flow cytometry |
| PE anti-mouse CD4 Antibody | Biolegend | 100407 | Flow cytometry |
| APC anti-mouse CD69 Antibody | Biolegend | 104514 | Flow cytometry |
| PE/Cyanine7 anti-mouse CD8a Antibody | Biolegend | 100721 | Flow cytometry |
| APC/Cyanine7 anti-mouse CD4 Antibody | Biolegend | 100413 | Flow cytometry |
| PE anti-mouse CD25 Antibody | Biolegend | 102008 | Flow cytometry |
| Alexa Fluor 647 anti-mouse/rat/human FOXP3 Antibody | Biolegend | 320014 | Flow cytometry |
| PE/Cyanine7 anti-mouse IFN-γ Antibody | Biolegend | 505826 | Flow cytometry |
| FITC anti-mouse IL-17A Antibody | Biolegend | 506908 | Flow cytometry |
| PE/Cyanine7 anti-mouse NK-1.1 Antibody | Biolegend | 108714 | Flow cytometry |
| PE anti-mouse IFN-γ Antibody | Biolegend | 505808 | Flow cytometry |
| APC anti-human/mouse Granzyme B Recombinant Antibody | Biolegend | 372203 | Flow cytometry |
| FITC anti-mouse/human CD11b Antibody | Biolegend | 101206 | Flow cytometry |
| PE anti-mouse Ly-6C Antibody | Biolegend | 128008 | Flow cytometry |
| PE/Cyanine7 anti-mouse F4/80 Antibody | Biolegend | 123114 | Flow cytometry |
| APC anti-mouse Ly-6G Antibody | Biolegend | 127613 | Flow cytometry |
| PE anti-mouse I-A/I-E Antibody | Biolegend | 107608 | Flow cytometry |
| APC anti-mouse CD11c Antibody | Biolegend | 117310 | Flow cytometry |
| PE/Cyanine7 anti-mouse CD80 Antibody | Biolegend | 104734 | Flow cytometry |
| FITC anti-mouse CD86 Antibody | Biolegend | 105005 | Flow cytometry |
| APC anti-mouse CD206 (MMR) Antibody | Biolegend | 141708 | Flow cytometry |
| PE anti-mouse TCR γ/δ Antibody | Biolegend | 118108 | Flow cytometry |
| FITC anti-mouse CD19 Antibody | Biolegend | 115506 | Flow cytometry |
| PE/Cyanine7 anti-mouse/human CD45R/B220 Antibody | Biolegend | 103221 | Flow cytometry |
| PE anti-mouse CD185 (CXCR5) Antibody | Biolegend | 145503 | Flow cytometry |
| PE anti-mouse IL-4 Antibody | Biolegend | 504103 | Flow cytometry |
| APC anti-mouse TNF-α Antibody | Biolegend | 506307 | Flow cytometry |
| Phospho-STING (Ser365) (D8F4W) Rabbit mAb | CST | 72971 | WB |
| Phospho-TBK1/NAK (Ser172) (D52C2) Rabbit mAb | CST | 5483 | WB |
| TBK1/NAK (E8I3G) Rabbit mAb | CST | 38066 | WB |
| Phospho-IRF-3 (Ser396) (4D4G) Rabbit mAb | CST | 4947 | WB |
| IRF-3 (D83B9) Rabbit mAb | CST | 4302 | WB |
| Phospho-NF-κB p65 (Ser536) (93H1) Rabbit mAb | CST | 3033 | WB |
| NF-κB p65 (D14E12)  Rabbit mAb | CST | 8242 | WB |
| STING (D2P2F) Rabbit mAb | CST | 13647 | WB |
| β-Actin (8H10D10) Mouse mAb | CST | 3700 | WB |
