# Supplementary material for: Antiviral and immune modulatory activities of STING agonists in a mouse model of persistent hepatitis B virus infection
Source: PLoS Pathog. 2025 Dec 9;21(12):e1013709. doi: 10.1371/journal.ppat.1013709 (PMC12700435; doi:10.1371/journal.ppat.1013709)
Supplement: S2 Table — (DOCX) [file ppat.1013709.s011.docx]

**Table S2. PCR primer sequences.**

| **Primer name** | **Primer Sequence** |
| --- | --- |
| murine IFNβ F | AGCTCCAAGAAAGGACGAACAT |
| murine IFNβ R | GCCCTGTAGGTGAGGTTGATCT |
| murine IFNα F | CCTGTGTGATGCAACAGGTC |
| murine IFNα R | TCACTCCTCCTTGCTCAATC |
| murine TNFα F | CAGTTCTATGGCCCAGACCCT |
| murine TNFα R | CGGACTCCGCAAAGTCTAAG |
| murine IL-6 F | AACGATGATGCACTTGCAGA |
| murine IL-6 R | GAGCATTGGAAATTGGGGTA |
| murine IFIT1 F | CAGAAGCACACATTGAAGAA |
| murine IFIT1 R | TGTAAGTAGCCAGAGGAAGG |
| murine IFITM3 F | GCCTATGCCTACTCCGTGAAGT |
| murine IFITM3 R | GCCTGGGCTCCAGTCACAT |
| murine ISG15 F | CTGTACCACTAGCATCACTGTG |
| murine ISG15 R | GGTGTCCGTGACTAACTCCAT |
| murine Mx1 F | AGACTTGCTCTTTCTGAAAAGCC |
| murine Mx1 R | GACCATAGGGGTCTTGACCAA |
| murine Mx2 F | GAGGCTCTTCAGAATGAGCAAA |
| murine Mx2 R | CTCTGCGGTCAGTCTCTCT |
| murine OAS1 F | CTTTGATGTCCTGGGTCATGT |
| murine OAS1 R | GCTCCGTGAAGCAGGTAGAG |
| murine OAS3 F | TCTGGGGTCGCTAAACATCAC |
| murine OAS3 R | GATGACGAGTTCGACATCGGT |
| murine STAT1 F | TCACAGTGGTTCGAGCTTCAG |
| murine STAT1 R | GCAAACGAGACATCATAGGCA |
| murine CXCL10 F | CCAAGTGCTGCCGTCATTTTC |
| murine CXCL10 R | GGCTCGCAGGGATGATTTCAA |
| murine GAPDH F | CTCTGGAAAGCTGTGGCGTGATG |
| murine GAPDH R | ATGCCAGTGAGCTTCCCGTTCAG |
| HBV pgRNA F | GAGTGTGGATTCGCACTCC |
| HBV pgRNA R | GAGGCGAGGGAGTTCTTCT |
| HBV core DNA F | GGCTTTCGGAAAATTCCTATG |
| HBV core DNA R | AGCCCTACGAACCACTGAAC |
